# PairMap: An Intermediate Insertion Approach for Improving the Accuracy of Relative Free Energy Perturbation Calculations for Distant Compound Transformations

**DOI:** 10.1021/acs.jcim.4c01634

**Published:** 2025-01-13

**Authors:** Kairi Furui, Takafumi Shimizu, Yutaka Akiyama, S. Roy Kimura, Yoh Terada, Masahito Ohue

**Affiliations:** † Department of Computer Science, School of Computing, Institute of Science Tokyo, Yokohama 226-8501, Japan; ‡ Alivexis, Inc., Tokyo 105-0004, Japan; § Department of Computer Science, School of Computing, Institute of Science Tokyo, Tokyo 152-8550, Japan

## Abstract

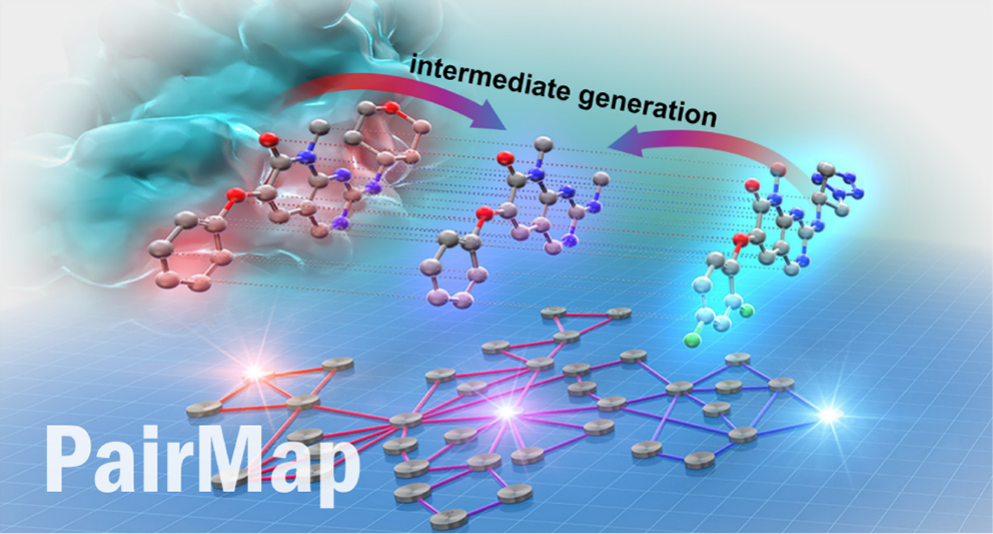

Accurate prediction of the difference in binding free energy between compounds is crucial for reducing the high costs associated with drug discovery. Relative binding free energy perturbation (RBFEP) calculations are effective for small structural changes; however, large topological changes pose significant challenges for calculations, leading to high errors and difficulties in convergence. To address such issues, we propose a new approach—PairMap—that focuses on introducing appropriate intermediates for complex transformations between two input compounds. PairMap-generated intermediates exhaustively, determined the optimal conversion paths, and introduced thermodynamic cycles into the perturbation map to improve accuracy and reduce computational cost. PairMap succeeded in introducing appropriate intermediates that could not be discovered by existing simple approaches by comprehensively considering intermediates. Furthermore, we evaluated the accuracy of the prediction of binding free energy using 9 compounds selected from Wang et al.’s benchmark set, which included particularly complex transformations. The perturbation map generated by PairMap achieved excellent accuracy with a mean absolute error of 0.93 kcal/mol compared to 1.70 kcal/mol when using the perturbation map generated by the conventional Flare FEP intermediate introduction method. Moreover, in a scaffold hopping experiment conducted with the PDE5a target involving complex transformations, PairMap provided more accurate free energy predictions than ABFEP calculations, yielding more reliable results compared to experimental data. Additionally, PairMap can be utilized to introduce intermediates into congeneric series, demonstrating that complex links on the perturbation map can be resolved with minimal addition of intermediates and links. In conclusion, PairMap overcomes the limitations of existing methods by enabling RBFEP calculations for more complex transformations, further streamlining lead optimization in drug discovery.

## Introduction

Precise estimation of the binding free energy between a given compound and its target protein is important to reduce the significant costs associated with drug design and lead optimization.^1−4^ Free Energy Perturbation (FEP) calculations^2−6^ have been garnering significant interest for the evaluation of candidate compounds in the early stages of drug development, as they can be used to evaluate the affinity of molecules with a useful level of precision of ±1 kcal/mol for a series.^7^ Two primary methodologies are recognized for FEP calculations: relative binding FEP (RBFEP),^2,8,9^ and absolute binding FEP (ABFEP).^10,11^ RBFEP is a method that determines the difference in the binding free energies between two compounds ΔΔ*G* by focusing only on different components of their structures. It has a relatively low computational cost and high accuracy when structural changes are small. However, RBFEP is prone to a high error rate and difficulties in convergence, especially when large-scale topological transformations are required between compounds. Large chemical changes render molecular simulations unstable, requiring high sampling costs and resulting in unreliable energy predictions. In contrast, ABFEP is a method that directly calculates the absolute binding free energy Δ*G* of a ligand, considering all degrees of freedom. ABFEP can directly calculate energy differences from a specific reference state, theoretically achieving high accuracy. Furthermore, it is particularly useful for evaluating the absolute energy of novel compounds. However, it is associated with a huge computational cost, and convergence is difficult due to the large perturbations required when coupling/decoupling the entire ligand. RBFEP calculations, which can significantly reduce computational cost and improve convergence by calculating ΔΔ*G* from known ligands, are currently popular in lead optimization campaigns.^4^ However, RBFEP calculations are limited in their application due to the drawback of large chemical changes between compounds that reduce the accuracy of these calculations and computational efficiency.^2,12−15^

To improve the accuracy and efficiency of free energy perturbation calculations in complex compound transformations, a variety of approaches exist. Grand Canonical Nonequilibrium Candidate Monte Carlo (GCNCMC)^16^ is a method that combines the traditional Grand Canonical Monte Carlo (GCMC)^17,18^ and Nonequilibrium Candidate Monte Carlo (NCMC) and helps accelerate the equilibration process by improving the sampling efficiency of the insertion and deletion of water molecules. Replica Exchange with Solute Tempering (REST2)^7^ is a popular approach that can increase sampling efficiency. The soft bond potential^19^ is also suggested to improve the core-hopping scenario. Other possible approaches include increasing the number of perturbation parameter λ intervals and increasing the simulation time, but these involve a trade-off between computational cost and accuracy.^12^

**Figure 1:**
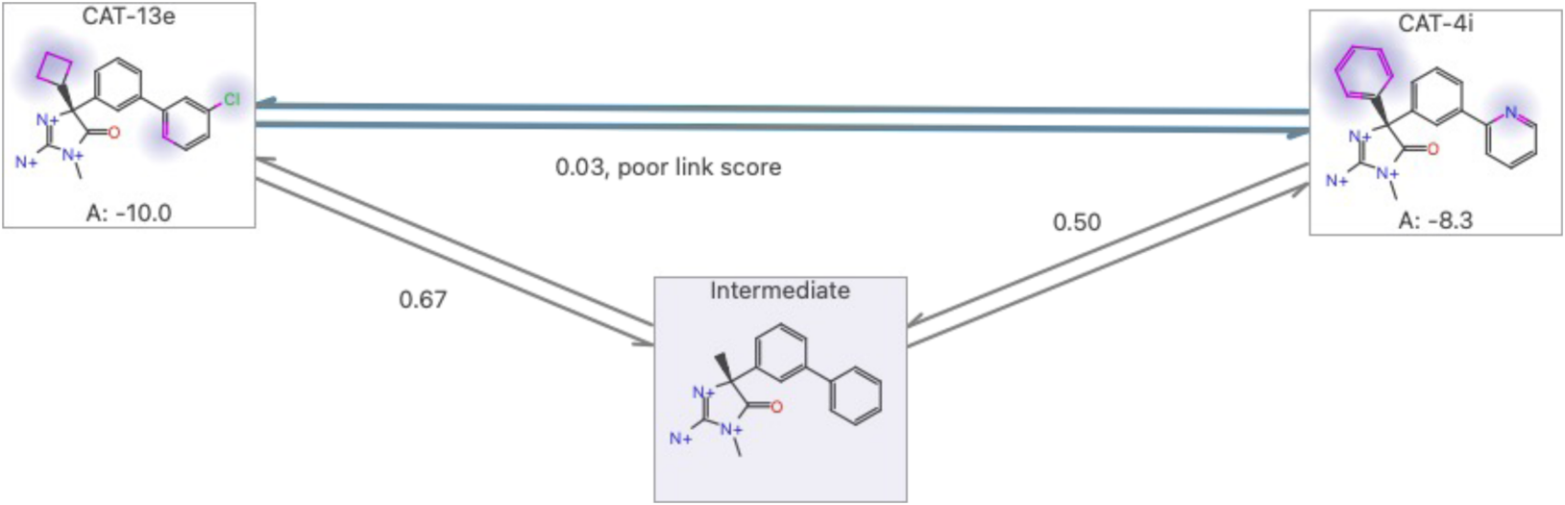
Example of the introduction of intermediates between compound pairs involving complex compound transformations in Flare FEP.

Another approach that is compatible with the other aforementioned methods to address this complex compound transformation involves dividing the transformation pathway and performing the transformation in smaller steps. The only existing approach based on this method is the intermediate introduction approach used in Flare FEP.^8,9^ Although the implementation method of this conventional approach is not publicly accessible, it is based on a simple approach of iteratively introducing derivative compounds called intermediates to links that are difficult to simulate on the perturbation map, a compound network that represents the experimental design.^20−22^ For example, as shown in Figure 1, introducing an intermediate between compounds with large transformations and performing ΔΔ*G* calculations via this intermediate can mitigate the overall difficulty of the calculation and improve accuracy and efficiency. However, although the Flare FEP approach can improve accuracy to a certain extent,^8^ it does not exhaustively examine all possible intermediate combinations, and therefore, may overlook the optimal pathway. In Flare FEP, the decision to introduce intermediates is based on whether the score representing the difficulty of transformation for an edge falls below a threshold; hence, the threshold remains the only controllable variable. Therefore, frequently, instances are noted when the number of intermediates introduced is not optimal. Depending on the threshold, introducing too many redundant intermediates can increase computational cost without enhancing the accuracy, whereas introducing too few intermediates may not resolve the issues in the original perturbation map. Originally, Flare FEP’s automatic intermediate generation is fundamentally designed to introduce missing intermediates into a compound series to improve computational efficiency. As it considers only the simple removal of edges with low link scores, it is not suitable for enhancing accuracy by introducing intermediates between two compounds with complex transformations. In actual drug discovery campaigns, there is also a need to determine ΔΔ*G* of ligands with novel structures as a further expansion of the derivative, such as scaffold hopping.^19,23,24^ Therefore, a different approach is needed to effectively select optimal intermediates and construct efficient transformation paths for complex transformations between two compounds.

In this study, we propose a novel method called PairMap that can be used to construct a perturbation map considering intermediate introduction for more complex transformations than previously possible. Unlike conventional Flare FEP’s automatic intermediate generation, PairMap focuses not only on improving computational efficiency by introducing intermediates into the derivative series, but on improving accuracy by introducing appropriate intermediates between two input compounds involving complex transformations. The purpose of this study was to construct a perturbation map that balances prediction accuracy and computational cost to perform ΔΔ*G* calculations. Specifically, PairMap efficiently performs compound transformations via the following three main steps: First, it exhaustively generates a set of candidate intermediates that can decompose a challenging compound transformation into smaller, more manageable substeps. This reduces the complexity of the free energy calculations at each perturbation step, thereby reducing potential convergence and sampling problems. Next, considering all generated intermediates, PairMap identifies an “optimal path” between input compounds. By “optimal,” we refer to a minimal yet effective combination of intermediates that both maintains a high level of calculation accuracy and avoids introducing unnecessary additional compounds or links. In practice, this means selecting the smallest set of intermediates that sufficiently break down complex transformations into simpler steps while minimizing additional computational costs. Finally, after determining the optimal path, PairMap constructs the perturbation map and performs the RBFEP calculations. Another feature of PairMap is the incorporation of thermodynamic cycles into the perturbation map by further introducing intermediates. Thermodynamic cycles play an important role in achieving high accuracy in compound transformations by correcting calculation errors based on the law of conservation of energy.^25^ Although Flare FEP also considers thermodynamic cycles, it does not distinguish between intermediates and nonintermediates, which sometimes results in the construction of nonoptimal maps. PairMap enables high-accuracy energy predictions by appropriately balancing intermediates and thermodynamic cycles.

## Materials and Methods

This section describes the algorithm of PairMap. In the following section, we will explain the details of each process of PairMap, which was used to construct a perturbation map between the compound pair, ligand A and ligand B for calculating ΔΔ*G*. The methodology of PairMap is divided into three processes: generation of diverse intermediates, determination of optimal intermediate paths, and construction of perturbation maps. Here, we considered the following requirements that a perturbation map must meet as much as possible when solving complex compound transformations by introducing intermediates.

**Figure 2:**
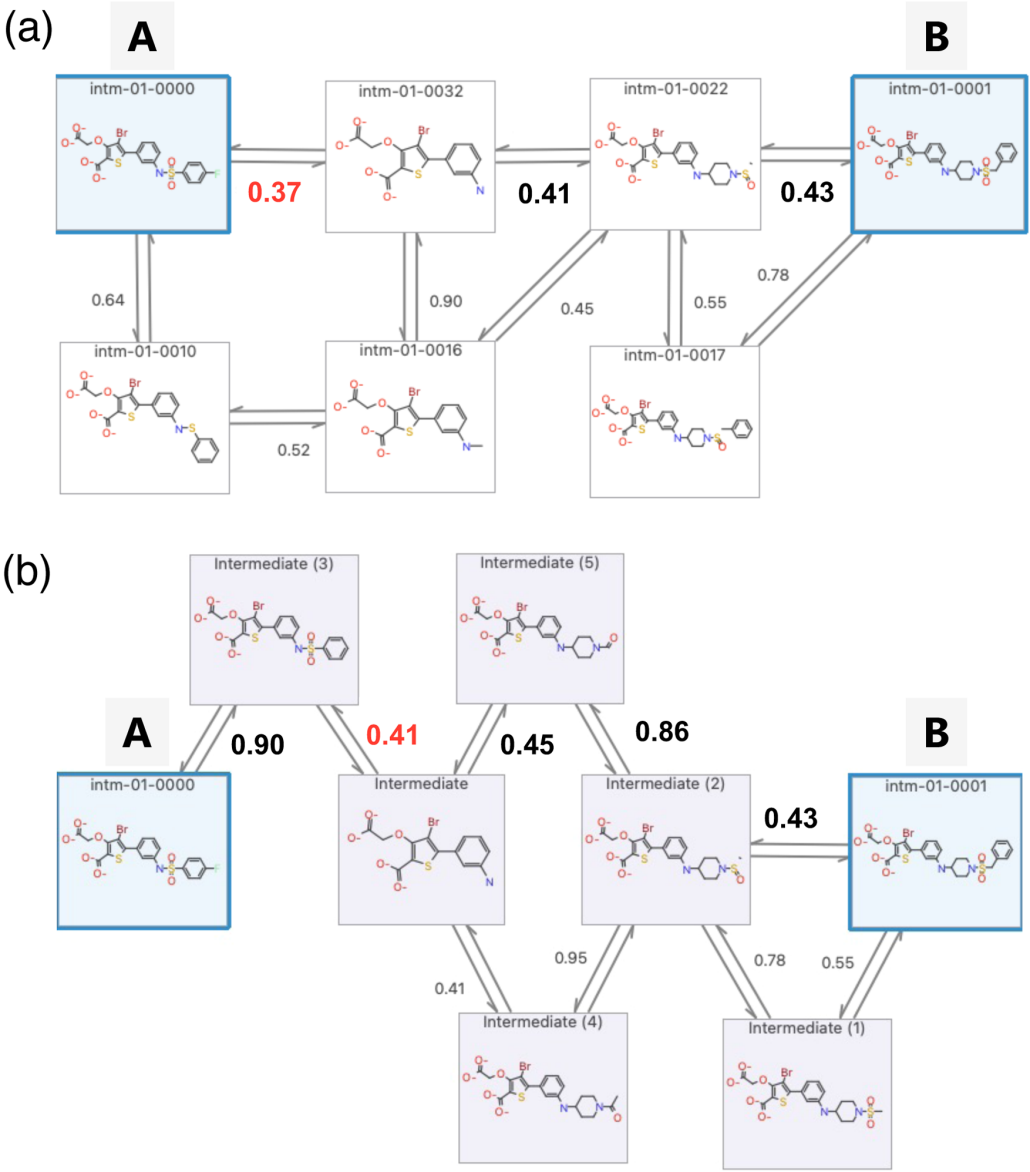
Example of perturbation map generation . The large text on the edges indicates the link scores in the shortest  path, with the red-highlighted score indicating the lowest link score of the shortest path. (a) is the output map obtained using PairMap. The shortest  path length is 3, and although the lowest link score in this path is 0.37, it meets the cyclicity (requirement 3). (b) is the output map obtained using Flare FEP (intermediate generation threshold 0.6). Graph (b) does not satisfy requirement 1, which requires that the  path must be shorter, because the minimum distance from ligand A to ligand B is 5, which is longer than in graph (a), even though the lowest link score is 0.41 in the shortest path, which is only a slight improvement on graph (a). In addition, the link with the lowest score in the shortest path of graph (b) is not included in any of the cycles, despite its low score of 0.41, and does not meet the cyclicity of requirement 3.

**Requirement 1:** The distance from ligand A to ligand B on the graph should be as short as possible (hereafter, distance refers to the length of the path from ligand A to ligand B on the graph). Requirement 1 serves as the fundamental principle for designing the  path, though it may need to be balanced with other requirements. Lomap, a key perturbation map generation tool, constrains the distance between active compounds to ensure it does not exceed a distance threshold value.^21^ This is because an increase in the number of links leads to a higher accumulation of errors in FEP calculations. In PairMap, calculating ΔΔ*G* accurately between input ligands A and B is most essential, making the distance from ligand A to ligand B on the graph shorter is corresponding to Lomap’s distance constraint. As shown in Figure 2b, if too many links are traversed between input ligands A and B, estimation errors will accumulate. Therefore, only the minimum necessary intermediates should be introduced, whereas complex transformations should be prevented.

**Requirement 2:** It is possible to reach from ligand A to ligand B without traversing through links that are difficult to simulate due to their complex transformations. The difficulty of a transformation is distinguished by a similarity score, called a link score, which will be described later. When selecting intermediates, we choose those that maximize the link scores on the edges of the optimal path. Requirements 1 and 2 can be seen as a trade-off: while shorter paths for requirement 1 reduce the accumulation of errors, ensuring that the path does not involve complex transformations for requirement 2 may require introducing additional intermediates.

**Requirement 3:** The output graph should avoid bridges, i.e., links that are not included in any of the cycles. This requirement ensures statistical reliability by introducing thermodynamic cycles,^25^ similar to Lomap’s cycle constraint.^21^ In the example of Figure 2b, the  path has bridges and needs to traverse difficult links; hence, these links cannot be used to correct errors or ensure statistical reliability using thermodynamic cycles. Therefore, the removal of edges and intermediates that violate this requirement should be avoided.

**Requirement 4:** The output graph has few calculation links and has as high a link score as possible. The purpose of this requirement is to reduce the computational cost by removing redundant edges while satisfying the thermodynamic cycle constraint of the  path in requirement 3. Requirement 4 can be satisfied by removing edges in the order of lowest link scores, as long as the other constraints are satisfied, similar to Lomap.^21^ However, although Lomap considers cycle constraints for all compounds, PairMap only needs to consider cycle constraints for the  path. Therefore, constraints other than those used in the Lomap method were used. In addition, unlike the Lomap method, the input compounds include intermediates that are unnecessary for the output map; hence, extraction of only the intermediates needed for constructing the perturbation map is required. Therefore, when constructing a perturbation map, only the desired intermediates among the available intermediates should be extracted.

Requirements 1, 2, and 3 pertain to prediction accuracy, whereas requirement 4 is intended to reduce the cost of FEP calculations by preventing redundant computations. To produce a result that best balances these requirements, PairMap generated perturbation maps using the following procedure. First, during the exhaustive generation step, all possible candidate intermediates were produced. Next, in determining the optimal intermediate path, the  path that satisfies requirements 1 and 2 was selected from among all combinations of the generated intermediates. Finally, considering requirement 4, a perturbation map connecting ligand A and ligand B was generated while maintaining alignment with requirement 3.

### Exhaustive Intermediate Generation

**Figure 3:**
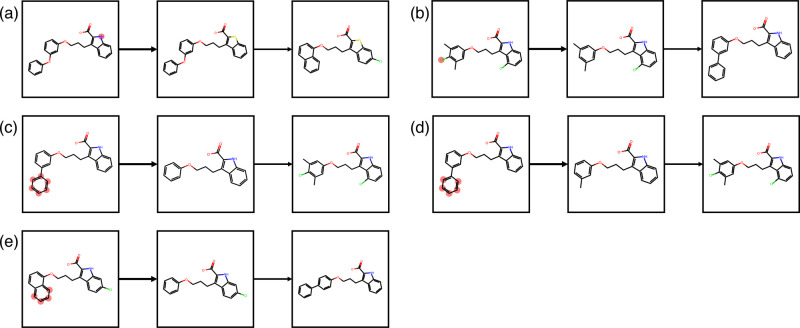
Intermediate generation operations. (a) Example of changing an atom within the MCS to bring it closer to the target compound. (b) Example of deleting an atom outside the MCS to bring it closer to the MCS. (c) Example of deleting a ring outside the MCS to bring it closer to the MCS (replacing it with a hydrogen atom). (d) Example of deleting a ring outside the MCS to bring it closer to the MCS (replacing it with a methyl group). (e) Example of deleting a section of a fused ring to bring it closer to the MCS.

In an intermediate generation, we considered the starting ligand A, the target ligand B, and the maximum common substructure (MCS), which accepts incomplete atomic type matching between the two input ligands A and B. The generated intermediates matched the atoms in the MCS part of ligand A that were not shared with the target ligand B and brought them closer to the target compound by deleting atoms in the non-MCS regions of ligand A (Figure 3). The operations for generating intermediates can be divided into the following four procedures:

**For atoms within the MCS**
  **Procedure a** Modify the atom to match the atom in the target compound.
**For atoms outside the MCS**
  **Procedure b** Delete one atom to bring it closer to the MCS compound.
  **Procedure c** Delete a ring and replace the deleted site with a hydrogen atom.
  **Procedure d** Delete a ring and replace the deleted site with a methyl group.
  **Procedure e** Delete a section of a fused ring.

**Figure 4:**
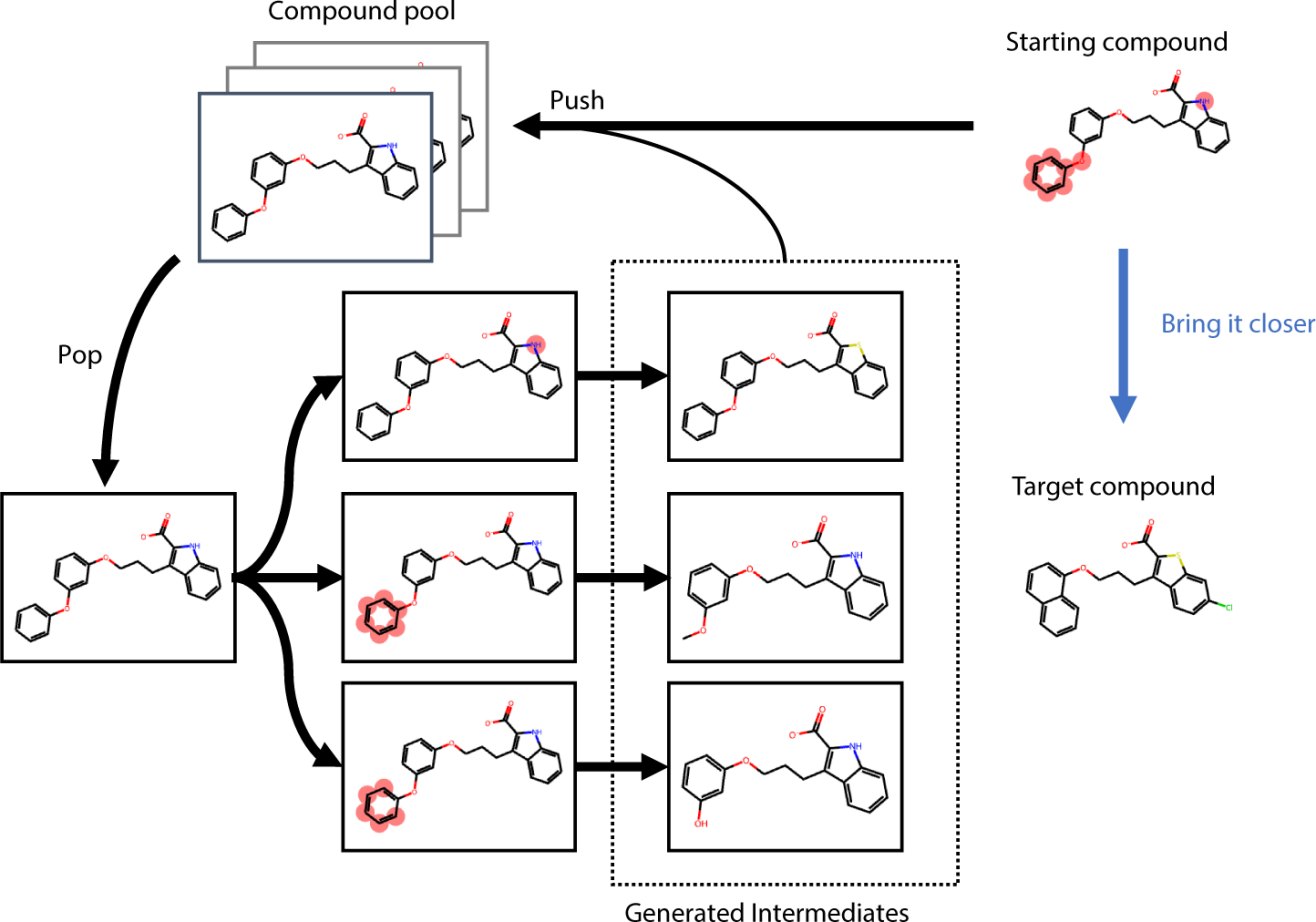
Overview of exhaustive intermediate generation.

Here, exhaustive intermediate generation is implemented by recursively repeating the above intermediate generation steps. This process employs a list called the compound pool to store the intermediates. Based on the push operation to store generated intermediates and the pop operation to get out intermediates to generate new intermediates, the generation is repeated until the compound pool is empty. The more detailed procedure of exhaustive intermediate generation is described as follows (Figure 4).

(1) Push the starting compound into the compound pool.
(2) Recursively perform the following processing steps until the compound pool becomes empty.
  (a) Pop one compound from the compound pool.
  (b) Generate intermediates by performing all possible operations among the intermediate generation operations. If multiple intermediates can be generated, then that many new intermediates will be generated .
  (c) Push only the newly generated intermediates into the compound pool.

By repeating this intermediate generation operation, the final intermediate compounds matched the MCS section of the target ligand B. The compounds generated from ligand A can be considered as a transformation path to reach the MCS of target ligand B. At this time, if ligand A is used as the starting compound, only intermediates that bring ligand A closer to the MCS compound will be generated. Hence, the direction of generation was reversed, and the operation of bringing ligand B closer to the MCS compound as the starting compound was also performed. At this point, we set a constraint implying that the net charges of the input two input compounds must be the same. Intermediates with charges different from those of input ligands A and B were excluded from further processing because using them as intermediates incurred additional computational costs. For the charge calculation of these intermediates, protonation was performed using the ligand preparation of Cresset Flare V7.2.^9^ In the next section, we consider the easing of complex transformations by selecting a minimum number of intermediates from these transformation pathways.

### Optimal Intermediate Path

Next, we determined the intermediate path that satisfied requirements 1 and 2 of the perturbation map generation from the generated group of intermediates. To consider the appropriate path, we defined an indicator called the link score^21^ that represented similarity between two compounds (the higher, the more similar). Lower link score reflects the complexity of compound transformations in FEP.

The link score was calculated based on the MCS which accepts incomplete atom type matching of the compound pair using the following formula: The score for an edge *e* between compounds A and B was defined as follows.
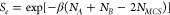
1where *β* is a parameter for scaling the change in link, and *N*_*A*_, *N*_*B*_, and *N*_*MCS*_ represent the numbers of heavy atoms in the two input compounds and the MCS, respectively. The term *N_A_* + *N_B_* − 2*N_MCS_* represents the total number of atoms that need to be inserted or deleted during the perturbation. Here, the *β* value was set to Lomap’s default of 0.1. The score was defined between 0 and 1, reaching a maximum value of 1 when the two compounds were identical. Next, as a measure of path desirability, we defined the path score *S*_*P*_ of the path  from A to B as follows:
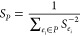
2In other words, the value represented the harmonic mean of the squares of the link scores *S*_*e*_ of the edges contained in the path, divided by the length of path *P*. A higher score *S*_*P*_ indicated a better path. Paths with this higher path score contain no bad edges that satisfy requirement 2. However, the process of simply selecting the path with the largest path score tends to favor longer edges as better edges, failing to satisfy the constraint of requirement 1, which is to maintain a short path length. Therefore, to limit the scope of paths using thresholds, the optimal path was selected as the path with the highest path score among the combinations of links with link scores above MIN_SCORE that can be obtained from compound  within the threshold MAX_DIST. These thresholds ensured that the output path was not too long and did not introduce bad links. The path obtained in this process was called the optimal intermediate path. Furthermore, compounds included in the optimal intermediate path were called optimal nodes, and links of the optimal path were called optimal links.

### Perturbation Map

In PairMap, a perturbation map with cycles connecting the two input compounds was constructed based on constraints, referencing the Lomap method.^21^ The conventional Lomap method removes unnecessary edges in the order of worst link scores based on constraints, assuming that all compounds will be used to construct the perturbation map. However, in PairMap, intermediates were introduced as auxiliary compounds to calculate only ΔΔ*G*_*FEP*_ between two input ligands A and B. Therefore, a procedure was required to eliminate unnecessary intermediates during the generation of the perturbation map to satisfy requirement 4, and PairMap used constraint conditions that differed from those of the Lomap. The procedure for generating a perturbation map in PairMap was as follows. First, link scores between all edges were calculated, and edges with link scores below a threshold were excluded during pruning. Next, the edges were sorted based on their scores, starting from the worst scores, and an attempt was made to remove edges in that order. Similar to that in Lomap, after removing an edge, if the constraints described below were satisfied, the removal was kept; otherwise, the graph was restored to its original state. Unlike Lomap, after removal of an optimal path edge, simple paths from ligand A to ligand B within the distance threshold MAX_SUBGRAPH_DIST were enumerated, and a subgraph of the set of nodes included in any of those paths was extracted before the constraints checks. If the subgraph extraction led to unmet constraints, the original graph was restored. This process of extracting subgraphs reduced the number of unnecessary intermediates in the final output and the number of constraint checks during the construction of the perturbation map. By performing this constraint check for all edges, it was possible to retain only the necessary and high-scoring edges. Furthermore, this check satisfied requirement 4 by retaining only the necessary edges with high link scores. The following two constraints were used:

(1) All optimal links must be retained.
  This was an essential condition for outputting the optimal path.
(2) All optimal links must be included in cycles of size less than or equal to the threshold MAX_CYCLE.
  This constraint ensured that all optimal links were included in the cycle, thereby satisfying requirement 3.
  However, since cycles which are too large lead to error accumulation, a threshold MAX_CYCLE was set.

Notably, this process was rendered more efficient by reducing the overall number of constraint checks for graphs obtained by the previously proposed approach.^22^

### Intermediate Introduction Perturbation Map for Congeneric Series

When performing FEP calculations for several dozen to several hundred compounds, it is common practice to plan experiments using perturbation maps for a series of compounds. In such cases, Flare FEP can avoid difficult transformations by constructing perturbation maps using the automatic intermediate generation feature when edge link scores fall below a specified threshold. However, as shown in Figure S7, a perturbation map with a Flare FEP with using a link score threshold of 0.6 resulted in many edges due to the introduction of redundant intermediates, which increased the computational cost. Additionally, it was noted that several parts where cycles consisting only of intermediates were formed; however, such cycles can improve accuracy, they should be carefully considered when targeting a large number of derivative series due to their significant computational cost. Such issues can be attributed to the fact that Lomap does not distinguish between intermediates and nonintermediates in its constraint checks.

Therefore, we also proposed a new method for constructing perturbation maps with intermediates by introducing intermediates using optimal paths generated by PairMap. First, a perturbation map was constructed using usual Lomap. Here, for all edges with link scores below the threshold, optimal intermediate paths were added to the input compounds using the PairMap method, excluding duplicates. Then, a new perturbation map was constructed using the Lomap method considering intermediates, using the original input compounds and the added intermediates. In this experiment, we modified the original Lomap constraints to not check connectivity, distance, and cyclicity for intermediates. Additionally, we excluded intermediates if they became disconnected from the main graph in the output graph or became leaf nodes without cycles. This process was performed because, in the intermediate introduction process, multiple similar intermediates may be introduced; however, to reduce calculation costs, only one of the necessary intermediates among them is sufficient. By repeating this process until no edges have link scores below the threshold, we can construct a perturbation map with optimal intermediates. However, to reduce computational costs, instead of reconstructing the perturbation map from scratch each time an intermediate is added, we only added edges between intermediates and existing nodes when reconstructing the perturbation map.

### Protocol for FEP

All FEP calculations in this study were performed using Flare FEP V7.2.^8,9^ The OpenFF version 2.0.0 force field^26,27^ was applied. Furthermore, the TIP3P^28^ explicit solvent water model was used, and AM1-BCC^29^ was employed for charge parameters.

The Flare FEP equilibration protocol was executed over a total duration of 500 ps through 5 sequential stages.^9^ In the first stage, the system underwent complete energy minimization. In the second stage, performed over 50 ps, the temperature was gradually increased to the target temperature in the NVT ensemble, during which all components except the solvent were kept under constraint. In the third step, performed over 100 ps, barostat was introduced while maintaining restraint on the protein and reducing restraint on the ligand. In the fourth stage, performed over 125 ps, the protein restraints were relaxed while maintaining backbone restraints and weak ligand restraints. In the final stage, performed over 225 ps, the backbone restraints were gradually reduced to complete the equilibration process. Each simulation step was performed with a time step of 4.0 fs along with hydrogen mass splitting factor setting of 1.50, followed by a 4 ns production run.

In FEP, the relative binding free energy is determined by gradually transforming the perturbation parameter λ from 0 to 1, and the number of divisions for this transformation is referred to as λ*s* hereafter. In Flare FEP, the number of λ*s* for each link is automatically decided at runtime based on the convergence of the computation, but the minimum λ*s* can be set as *min*λ. The *min*λ and whether the simulations for a compound pair were performed bidirectionally or unidirectionally differed for each experiment.

## Results and Discussion

### Introduction of Intermediates into Compound Transformations

First, we evaluated the effectiveness of the optimal intermediate path by PairMap and compared it to that of the existing automatic intermediate generation process in Flare FEP for common compound transformations. To evaluate the typical FEP use case, we extracted up to 5 unique compound pairs with a direct link score of 0.4 or less for each of 8 targets from Wang et al.’s data set.^7^ For a total of 31 compound pairs from 7 targets, excluding thrombin, we evaluated whether the introduction of intermediates would result in the worst link score of the  path exceeding the threshold of 0.5. Since the ligand for Thrombin has only slight transformation, introduction of intermediates was not necessary. For the automatic intermediate generation in Flare FEP, the threshold for intermediates was set to 0.5, and the shortest path among all combinations of generated compounds that exceeded the threshold of 0.5 with the highest worst link score was selected as the output. The perturbation map generated by Flare FEP cannot consider factors such as the path length between input compounds or the worst link. To ensure a fair comparison, this approach was used. For PairMap, the optimal path with MIN_SCORE = 0.5 and MAX_DIST = 4 was selected as the output, ensuring that the link score would always be 0.5 or higher. The desirability of a path is based on the following criteria: First, it must be able to create a path with a worst-link score of 0.5 or more. In addition, while a shorter path length is preferable, if the path lengths are the same, a higher worst link score is desirable. However, it should be noted that the shortest path and the worst link score exist in a trade-off relationship.

**Figure 5:**
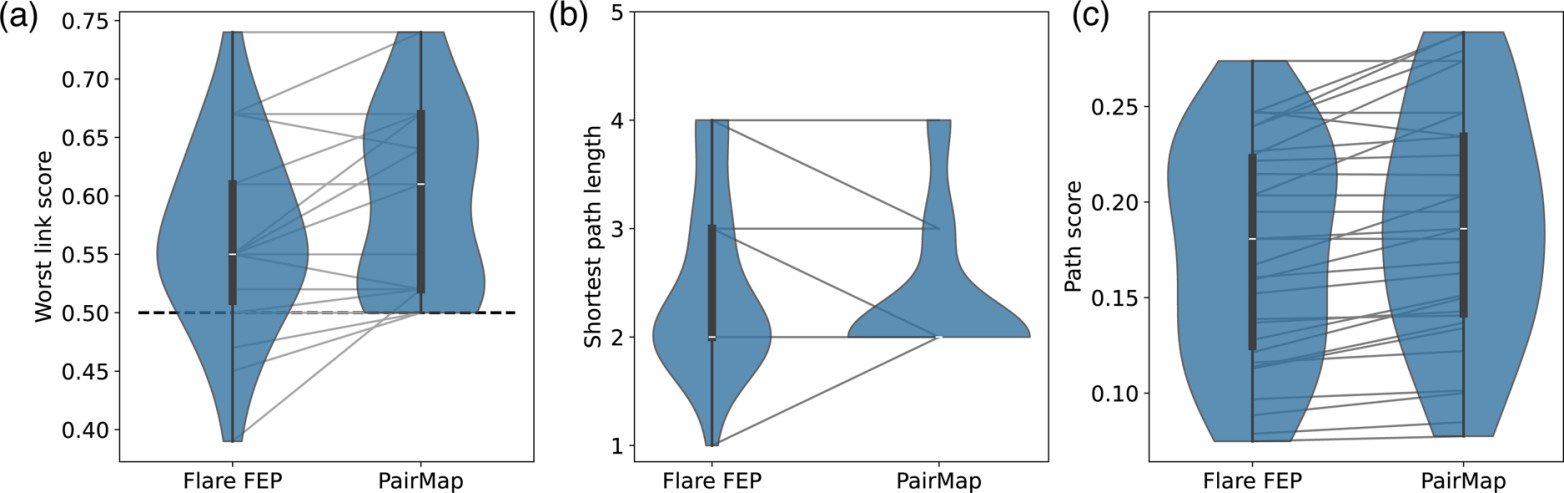
Distribution of (a) worst link score, (b) shortest path length, and (c) path score for Flare FEP's automatic intermediate generation and PairMap's optimal intermediate path.

Figure 5 shows the distribution of the worst link score, shortest path length, and path score for the intermediate path determined by Flare FEP’s automatic intermediate generation and PairMap’s optimal intermediate path for all selected compound pairs. First, in the case of Flare FEP’s automatic intermediate generation, the worst link score was below the threshold of 0.5 in some cases. Furthermore, the median worst link score of the optimal path by PairMap was approximately higher (better) by 0.05 than that obtained using the Flare method. Moreover, the most frequent value of the path distance for both methods was two; however, the frequency of this value was higher for the PairMap method than for the Flare method. These results suggest that while the optimal path by PairMap can find paths with the worst link scores of 0.5 or higher, in some cases, Flare FEP was unable to introduce such intermediates. The path score was higher for PairMap than for Flare FEP’s output in most cases; however, this finding is apparent because PairMap selected the one with the highest path score. The reason why Flare FEP’s method has a higher path score in some cases is that Flare FEP’s output includes paths outside the range of MIN_SCORE and MAX_DIST settings, and these path scores may be higher than that of PairMap’s. This suggests that it is necessary to set these thresholds, as sorting bypass score alone fails to provide the desired output.

**Figure 6:**
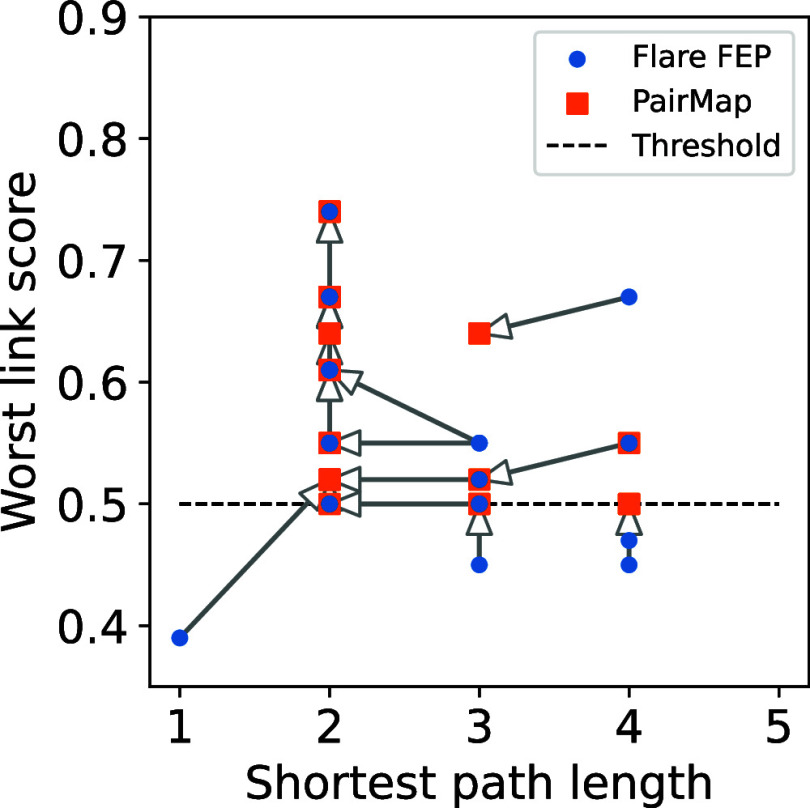
Relationship between shortest path length and worst link score for Flare FEP’s automatic intermediate generation and PairMap’s optimal intermediate path. The arrows indicate the correspondence between the same compound pairs, starting with Flare FEP’s method and ending with PairMap’s method. A left-pointing arrow indicates that PairMap had a shorter path length, and an upward-pointing arrow indicates that PairMap improved the worst-link score.

Figure 6 shows the relationship between the shortest path length and the worst link score for each method, with arrows connecting the scores from Flare FEP to PairMap's optimal path for the same compound pair. A left-pointing arrow indicates that PairMap's optimal path achieved a shorter path length, while an upward-pointing arrow shows that PairMap's optimal path obtained a higher (better) worst link score with the same path length. The right-pointing arrows only correspond to cases where Flare FEP could not introduce any intermediates (distance 1), and all other cases are associated with only left-pointing or upward-pointing arrows, indicating the absence of instances where the results worsened due to PairMap’s optimal path. Furthermore, 4 cases were noted for which Flare FEP’s method did not exceed the threshold of 0.5. Considering all possible combinations that can be generated by the exhaustive intermediate generation, paths equivalent to or better than the intermediates generated by Flare FEP can be constructed.

As described above, considering intermediates comprehensively is useful for introducing the minimum necessary intermediates that cannot be constructed by a greedy approach. In particular, the 4 cases for which Flare FEP could not raise the threshold to 0.5 or higher and the 6 cases for which shorter paths could be constructed indicate the limitations of Flare FEP’s simple intermediate introduction method.

### FEP Calculations of Complex Compound Transformations Using Wang-9 Data Set

**Figure 7:**
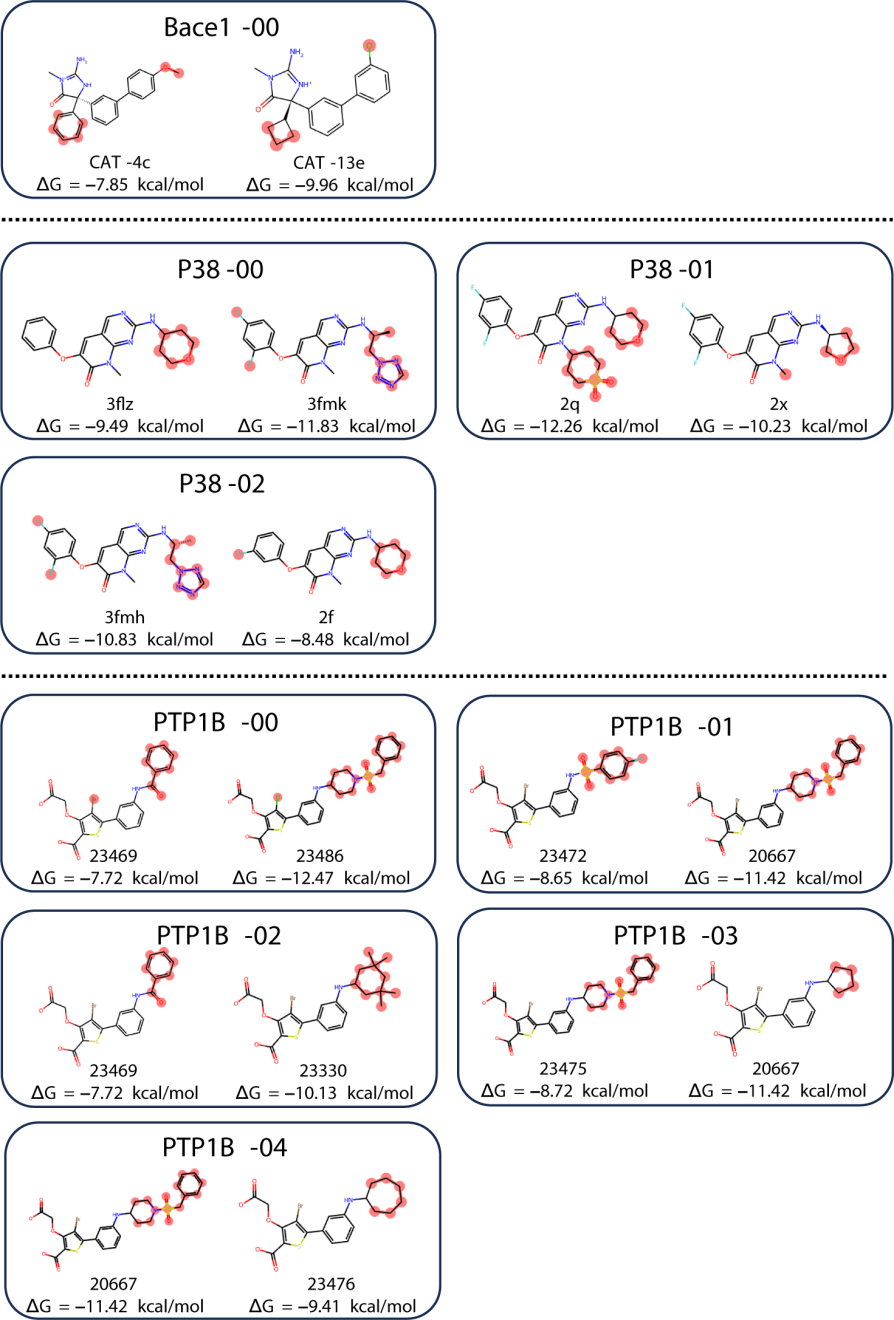
Breakdown of the Wang-9 data set. The compound pair name and experimental Δ*G* are shown.

To evaluate the performance of the proposed method PairMap, we constructed an intermediate introduction perturbation map for complex compound transformations and performed FEP simulations using the generated maps. Here, from Wang et al.’s^7^ data set that was used for evaluating general FEP performance on 8 targets, we selected 9 compound pairs that involved complex transformations, such as insertion or deletion of two or more rings and have experimental ΔΔ*G*_*exp*_ values of 1 to 2 kcal/mol or higher. The 9 selected compound pairs shown in Figure 7 were used as the Wang-9 data set for the evaluation using PairMap. The ligand-protein complex structures used for FEP calculations were obtained from a data set of a previous study^8^ and archived github repository.^30^

First, the effectiveness of intermediate paths and thermodynamic cycles by PairMap was compared in the following 5 settings:

**1-Hop:** A perturbation map using Flare FEP’s intermediate generation to introduce only one intermediate, with direct links and links via one intermediate.

**NoCycle:** A perturbation map using only the optimal intermediate path of PairMap.

**PairMap:** The final output map of PairMap, adding cycles to NoCycle.

**Flare(0.4/0.6):** Perturbation maps generated by Flare FEP’s automatic intermediate generation.

The values 0.4 and 0.6 in brackets represent the link score parameters, respectively, and Flare FEP generated intermediates and constructs perturbation maps that exceeded these values.

For perturbation map construction, the optimal path setting had a maximum distance of MAX_DIST = 3, the allowed link score was MIN_SCORE = 0.2, and the PairMap setting had the maximum cycle size that can be introduced as MAX_CYCLE = 4, with the maximum distance allowed for all  paths as MAX_SUBGRAPH_DIST = 4. However, for the optimal path length, we compared it with the settings of NoCycle(4) and PairMap(4), which determined the optimal path by fixing the path distance to 4. The case with the default of MAX_DIST = 3 was referred to as NoCycle(3), PairMap(3), or simply PairMap or NoCycle. For the Bace-01 compound pair, the same perturbation map was used as the distance of 2 and was sufficient.

FEP simulations were performed with unidirectional FEP, with *min*λ = 5 for NoCycle and PairMap, and 10 or more λ*s* for 1-Hop due to the difficulty of calculating the complex transformation. In the case of 1-Hop, direct computation of too complex transformations may eventually fail to compute ΔΔ*G*_*FEP*_. In such cases, ΔΔ*G*_*FEP*_ is calculated using only the links for which calculation results were obtained.

**Figure 8:**
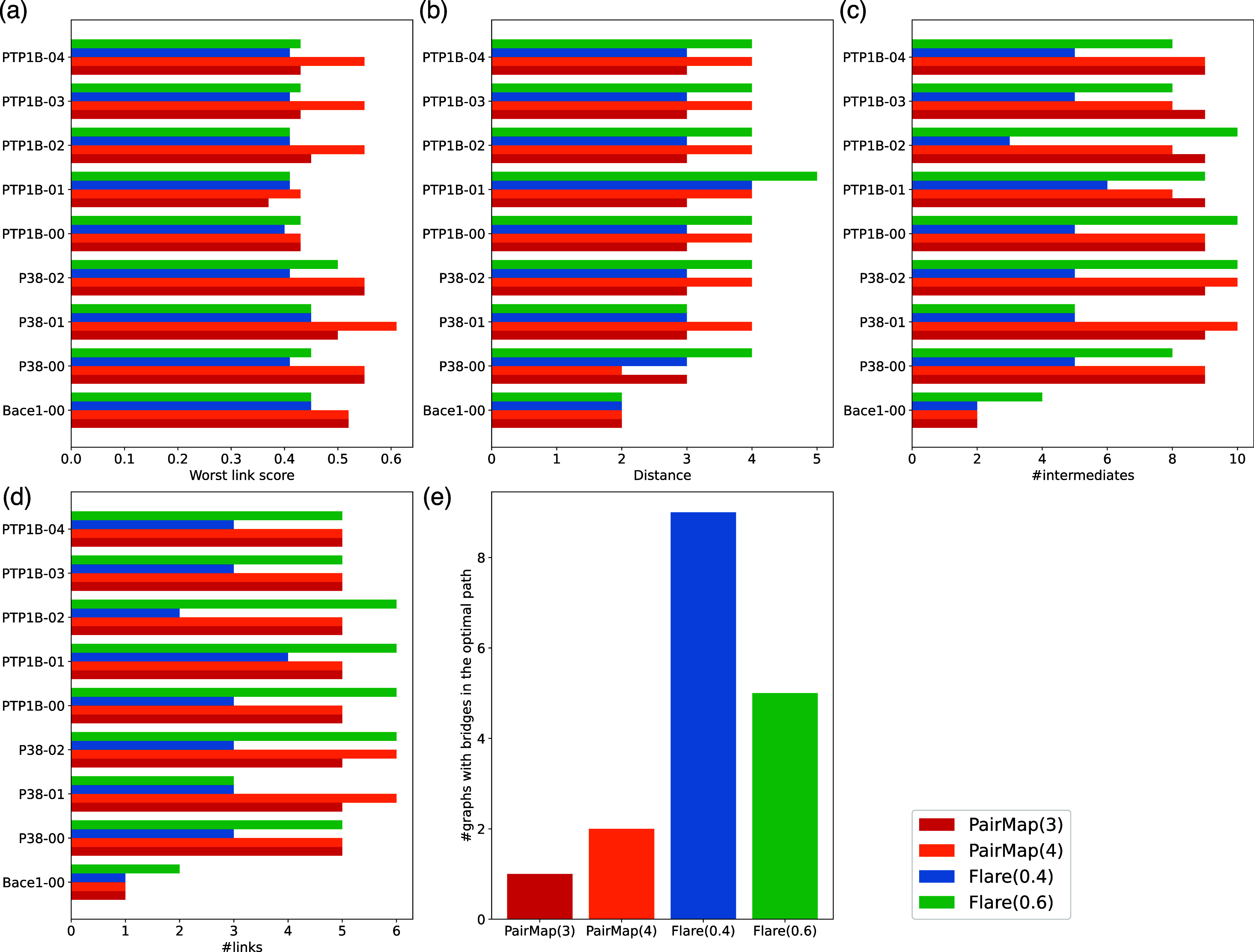
5 statistics of the output graph reflect, (a) the worst link score, (b) the distance between the input compounds, (c) the number of introduced intermediates, (d) the number of links in the output map, and (e) the number of graphs with bridges (where a bridge is defined as a link not included in any cycle) in the optimal path. Only the worst link score is better when larger, while the other metrics are better when smaller.

Figure 8 shows the statistics of the output graphs for the 4 intermediate introduction methods in the Wang-9 data set. First, in PairMap(3) and PairMap(4), the worst link scores for Bace1 and P38 were approximately 0.5 or higher. This value was higher than that obtained using Flare FEP’s intermediate introduction. In contrast, PTP1B demonstrated improvement to a level comparable to Flare’s method, but not as significant as enhancements observed with Bace1 or P38. This finding was hypothesized to reflect the difficulty of generating optimal paths for each target. Upon comparing PairMap(3) and PairMap(4), we observed that for the difficult cases of PTP1B, increasing the distance improved the worst link score. Next, upon examining the length of the optimal path, we observed that for PairMap with a fixed distance, only Bace1 had a distance of 2, which can be attributed to the fact that it is a compound pair for which the score can be improved with only one intermediate. Furthermore, from the total number of graphs containing at least one link that did not belong to any cycle (namely, forming a bridge) in the  path, PairMap constructed a path without a bridge in the optimal path in almost all cases, whereas Flare(0.4) included a bridge in the optimal path in all cases. Regarding the length of the optimal path in Flare(0.4), it was the same as MAX_DIST = 3 in PairMap. In contrast, in Flare(0.6), despite fewer cases in which the optimal path included a bridge, the average of the optimal path length value was higher, and extremely long perturbation maps with a distance of 5, such as PTP1B-01, were also generated. The aforementioned results suggest that the PairMap method produced perturbation maps considering the cyclicity, link scores, and length of the path between compound pairs. The number of links and intermediates used in each perturbation map was related to the cost of FEP calculations, and the PairMap method typically used more links and intermediates than Flare(0.4). Therefore, in terms of computational cost, Flare(0.4) had the lowest costs. Furthermore, when comparing PairMap(3) and PairMap(4), even though the length of the optimal intermediate path differed between the two methods, the number of intermediates and links used were almost the same. This was thought to be because both methods set MAX_SUBGRAPH_DIST = 4.

### FEP Calculation Results for Wang-9 Data Set

**Figure 9:**
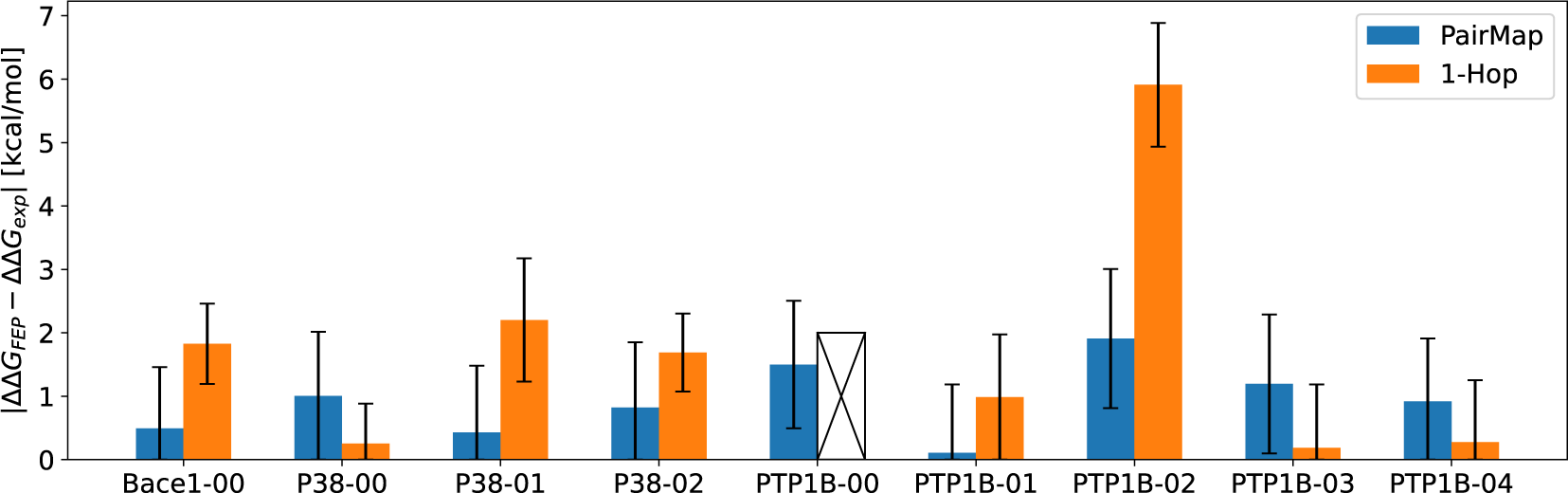
Results of ΔΔ*G*^*error*^ = |ΔΔ*G*_*exp*_ – ΔΔ*G*_*FEP*_| for the Wang-9 data set (comparison of PariMap and 1-Hop). A cross in the bar chart means that the ΔΔ*G* calculation failed. Error bars indicate 90% confidence intervals calculated by 10,000 bootstrap samplings.

Here, Figure 9 shows the ΔΔ*G*^*error*^ (the error between the experimental  and the FEP ) for the PairMap(3), and 1-Hop perturbation maps in the Wang-9 data set (Results for Δ*G* are shown in Figure S1). Note that the 1-Hop experiment for PTP1B-00 failed to calculate ΔΔ*G*_*FEP*_ for the compound pair; hence, the results are not shown. In the 1-Hop experiment for PTP1B-00, the calculation of ΔΔ*G*_*FEP*_ failed due to numerical instabilities caused by abrupt energy changes in certain λ windows. In Flare FEP, such numerical instabilities frequently occur when performing complex transformations, and in such cases, simple intermediate introduction methods like 1-Hop may be insufficient to achieve proper convergence. With 1-Hop, cases for which the prediction error was extremely large, such as PTP1B-02 were noted, along with cases where ΔΔ*G*_*FEP*_ could not be calculated, such as PTP1B-00. The direct ΔΔ*G*_*FEP*_ could be calculated by 1-Hop only for Bace1-00, P38-00, and P38-02, indicating that introducing multiple intermediates is essential for calculations involving complex transformations in Flare FEP. Therefore, when calculating ΔΔ*G*_*FEP*_ for complex compounds, it is desirable to introduce some intermediates.

**Figure 10:**
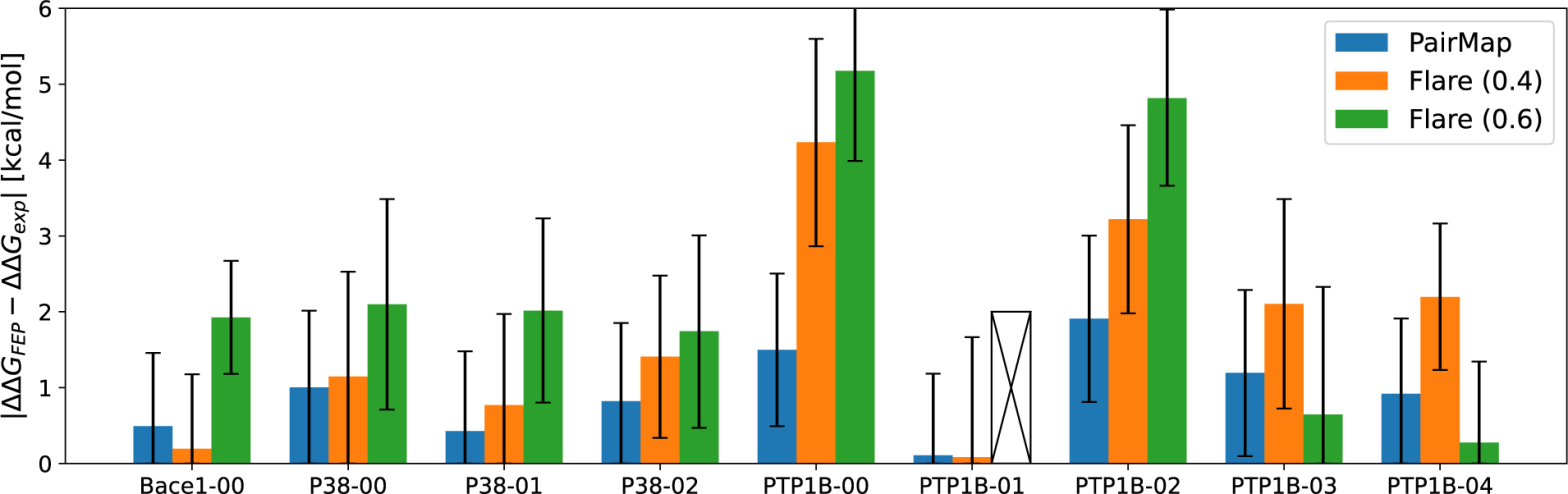
Results of ΔΔ*G*^*error*^ = |ΔΔ*G*_*exp*_ – ΔΔ*G*_*FEP*_| for the Wang-9 data set (comparison of PariMap and Flare’s automatic intermediate generation).

Figure 10 shows the ΔΔ*G*^*error*^ for Flare’s automatic intermediate generation and PairMap(3) in the Wang-9 data set (Results for Δ*G* are shown in Figure S2). Note that the result for Flare(0.6)’s PTP1B-01 was considered a failure because ΔΔ*G*_*FEP*_ was an extremely large outlier. The reason for this failure is that it contained a link with a significantly larger ΔΔ*G*. The presence of such an incorrect link in the path makes it impossible to obtain an accurate value even if the accuracy of the other links is good. Therefore, since too long paths increase the likelihood of link failures, requirement 1 is important. Flare(0.4) had low prediction accuracy for PTP1B, suggesting that introducing cycles is particularly important for complex compound pairs. Furthermore, Flare(0.6) showed large prediction errors, except for PTP1B-03 and PTP1B-04. This may be attributed to the large distances between input compounds and the paths containing bridges with low link scores.

### FEP Calculation Results for Wang-9 Data Set for Each PairMap Parameter

**Figure 11:**
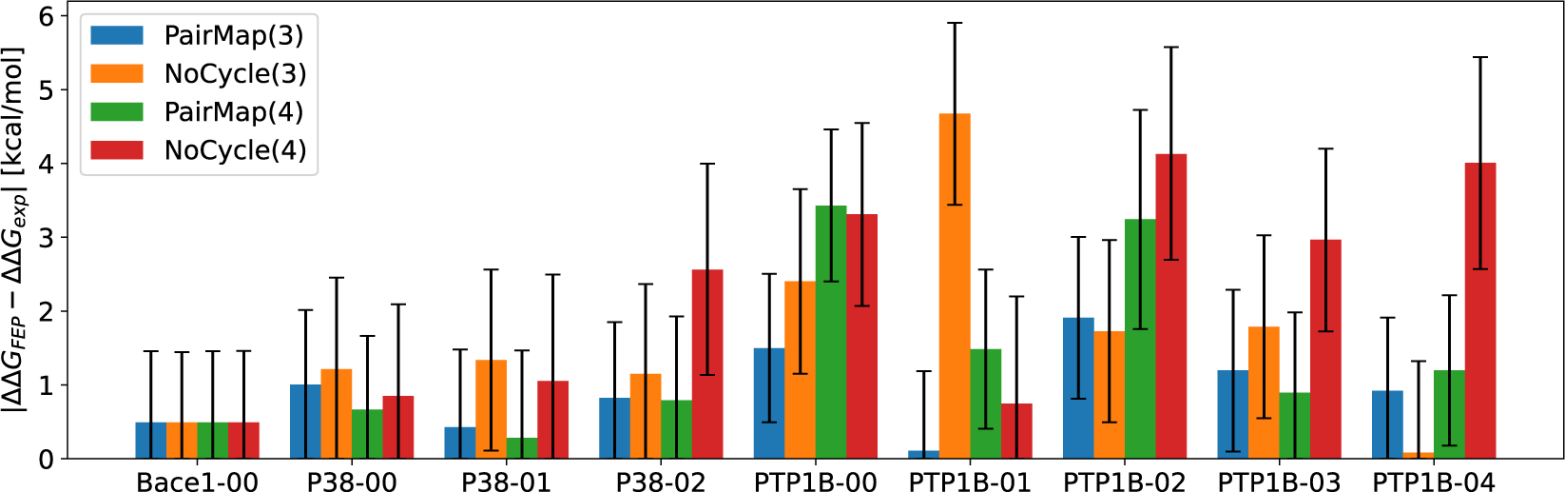
Results of ΔΔ*G*^*error*^ = |ΔΔ*G*_*exp*_ – ΔΔ*G*_*FEP*_| for the Wang-9 data set (distance 3 vs distance 4).

Figure 11 shows the ΔΔ*G*^*error*^ for PairMap(3), NoCycle(3), PairMap(4), and NoCycle(4) in the Wang-9 data set (Results for Δ*G* are shown in Figure S3). First, when comparing PairMap and NoCycle at both distances, NoCycle showed worse ΔΔ*G*^*error*^ in most cases, suggesting that the cyclicity requirement in PairMap contributed to reducing the prediction error. Furthermore, for PTP1B-01, NoCycle(3) depicted a high error, and ΔΔ*G*^*error*^ differed significantly from the PairMap(3) result. This finding can be attributed to the error correction by cycles, along with the canceling of the error of the optimal path and the error of the cycle; hence, it may be optimistic to interpret these results as an enhancement in accuracy attributed to the addition of cycles (Figure S4).

Furthermore, for PTP1B-00 and PTP1B-02, the prediction error was large for PairMap(4) and NoCycle(4). This finding can be attributed to error accumulation, similar to the case of Flare(0.6) where the distance between compounds was high. The aforementioned results further suggest that while too few intermediates, such as in 1-Hop, render performing reasonable calculations difficult, too long a distance, such as in PairMap(4) and NoCycle(4), does not lead to improved accuracy.

### Summary of FEP Calculation Results for Wang-9 Data Set

Table 1 shows the mean absolute error (MAE) between ΔΔ*G*_*exp*_ and ΔΔ*G*_*FEP*_ for all settings. The failed compound pairs of PTP1B-00 for 1-Hop and PTP1B-01 for Flare(0.6) were simply excluded from the calculation; hence, the performance should not be judged on these metrics only. When considering only the metrics, PairMap had the best results, NoCycle, 1-Hop, and Flare(0.4) were comparable, and Flare(0.6) had the worst results. Considering that 1-Hop comprised many failed links and required as many λ*s* as possible, NoCycle or Flare(0.4) was considered superior in terms of computational cost. However, it should be noted that a PairMap(3) MAE of less than 1 kcal/mol is an optimistic value since, as mentioned previously, it is not an essential improvement of PTP1B-01. Furthermore, despite NoCycle(4) having the second-largest MAE after Flare(0.6), PairMap(4) had the second-best prediction accuracy after PairMap(3). Therefore, introducing cycles to each link, which is a requirement of PairMap, is considered important for improving prediction accuracy. In conclusion, using the perturbation map generated by PairMap, accurate calculation of ΔΔ*G*_*FEP*_ can be performed for complex compound transformations.

**Table 1:** Mean Absolute Error (MAE) between ΔΔ*G*_*exp*_ and ΔΔ*G*_*FEP*_ for the Wang-9 Data Set (All Settings)^a^

	PairMap(3)	PairMap(4)	NoCycle(3)	NoCycle(4)	Flare(0.4)	Flare(0.6)	1-Hop
MAE (kcal/mol)	0.931 ± 0.347	1.386 ± 0.661	1.652 ± 0.800	1.970 ± 0.954	1.707 ± 0.800	2.337 ± 1.098	1.667 ± 1.185

a The 95% confidence interval was calculated using the bootstrap method (*N* = 1000).

### Execution Time of Map Construction on Wang-9 Data Set

**Figure 12:**
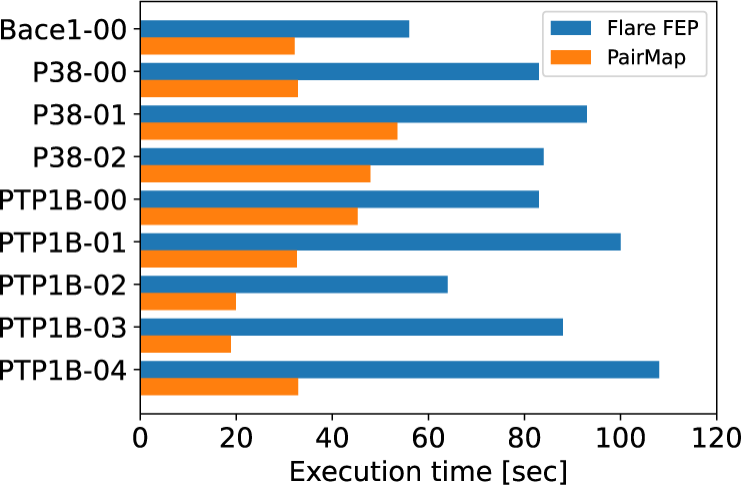
Execution time for automatic intermediate generation by Flare FEP and perturbation map construction by PairMap on the Wang-9 data set.

**Figure 13:**
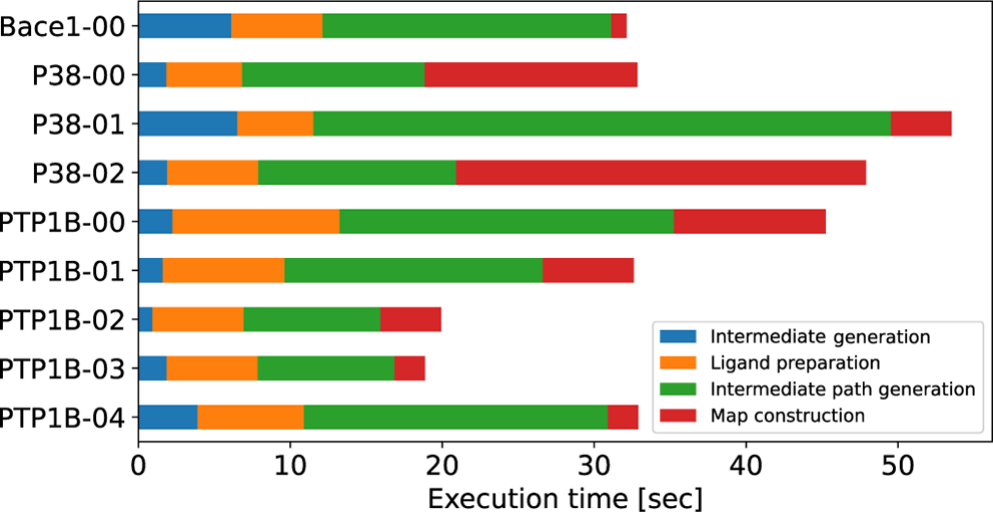
Breakdown of PairMap execution time on the Wang-9 dataset. The breakdown consists of four steps: intermediate generation, ligand preparation, intermediate path generation, and map construction.

We evaluated the practicality of our method in terms of execution time for optimal path construction and perturbation map generation. Figure 12 shows the time required for automatic intermediate generation by Flare FEP and perturbation map construction by PairMap for each compound pair in the Wang-9 data set. As shown in the figure, PairMap constructs perturbation maps faster than Flare FEP for all compound pairs. This is because Flare FEP’s automatic intermediate introduction iteratively performs perturbation map construction, whereas PairMap comprehensively generates intermediates in a single calculation and constructs the perturbation map only once. Therefore, PairMap is more convenient than existing methods in terms of execution time as well. Additionally, Figure 13 shows the breakdown of PairMap’s execution time. Note that map construction is not necessary if only optimal intermediate paths need to be constructed. As shown in the figure, the intermediate path generation process takes the most time in most cases. The calculation of link scores increases quadratically with the number of intermediates due to score matrix computation. For more challenging situations, pruning the number of intermediates used should be considered. Furthermore, when constructing perturbation maps for a series of compounds, intermediate path generation needs to be performed multiple times. When dealing with extremely large and diverse compound series, the number of links requiring intermediates may increase, and it should be noted that execution time may increase linearly with the number of required intermediates.

### FEP Calculation Cost for Wang-9 Data Set

Table 2 shows the total λ*s* of the perturbation maps for all experiments, i.e., the associated computational costs. Flare(0.6) depicted the highest computational cost, followed by PairMap (for distances 3 and 4), Flare(0.4), and the remaining NoCycle (for distances 3 and 4) and 1-Hop, which exhibited similar computational costs. While 1-Hop had a low computational cost in terms of numbers, it was at least as expensive as NoCycle because λ*s* for failed links were excluded. NoCycle(3) should also be used because the 1-Hop calculation is unstable and may not converge. In the case of PairMap and NoCycle, the computational cost was the same across distances. This was likely assumed because shorter distances typically render convergence of calculations difficult, with a requirement of more λ*s*. Hence, PairMap(3) was preferable for calculation accuracy, whereas NoCycle(3) was preferable for low-cost calculations.

**Table 2:** Total λ*s* in FEP Calculations for the Wang-9 Data Set

	PairMap(3)	PairMap(4)	NoCycle(3)	NoCycle(4)	Flare(0.4)	Flare(0.6)	1-Hop
Bace-00	31	31	13	13	21	43	35
P38-00	63	64	24	24	37	61	33
P38-01	69	75	24	29	57	57	24
P38-02	66	81	25	32	37	76	34
PTP1B-00	75	69	33	28	47	108	-
PTP1B-01	79	79	33	39	56	112	29
PTP1B-02	76	64	33	30	30	84	24
PTP1B-03	75	70	29	29	44	100	23
PTP1B-04	79	76	30	33	45	75	25
Total λ*s*	613	609	244	257	374	716	>227

### FEP Calculation for Merck Data Set

**Figure 14:**
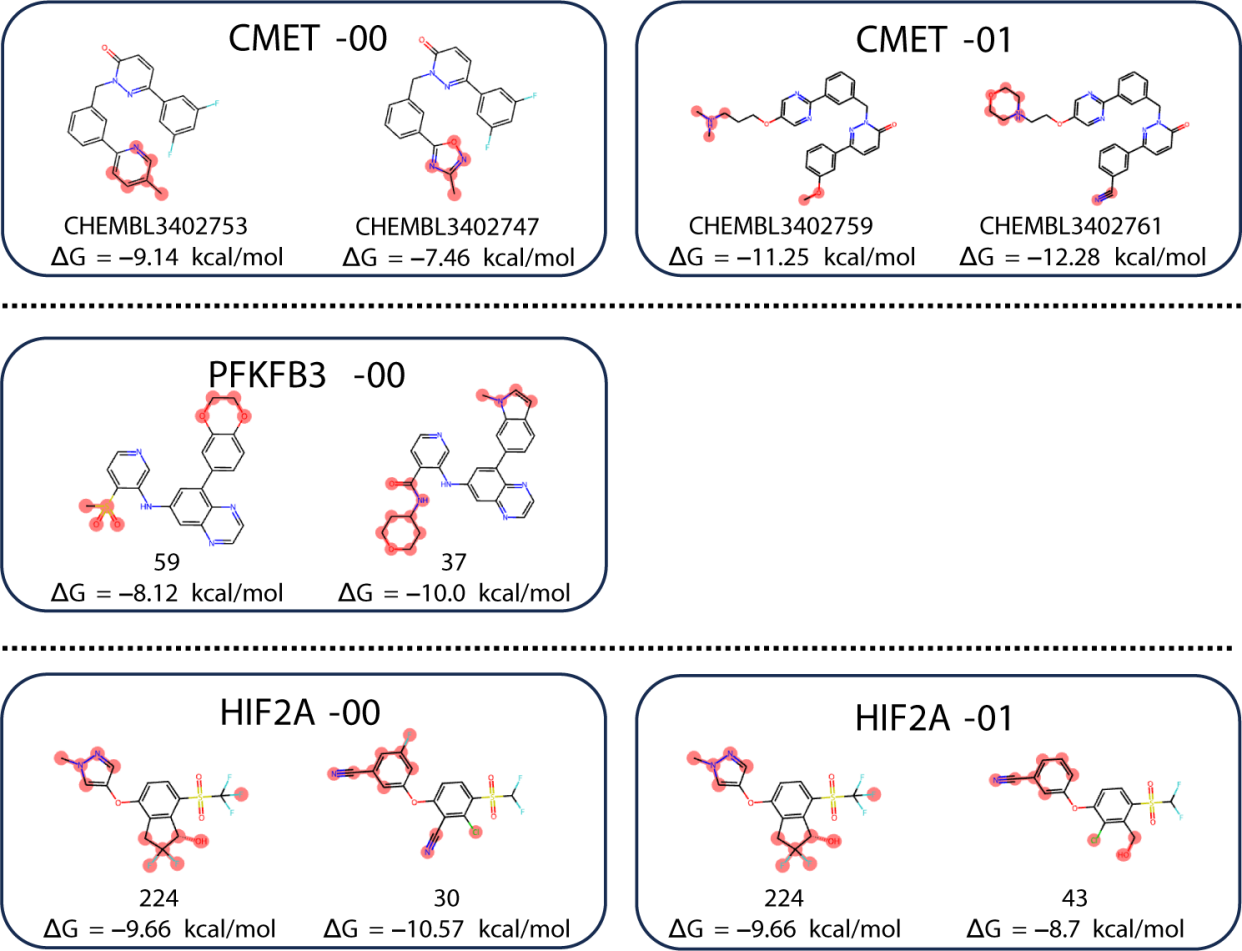
Breakdown of the selected compound pairs from Merck data set. The compound pair name and experimental Δ*G* are shown.

**Figure 15:**
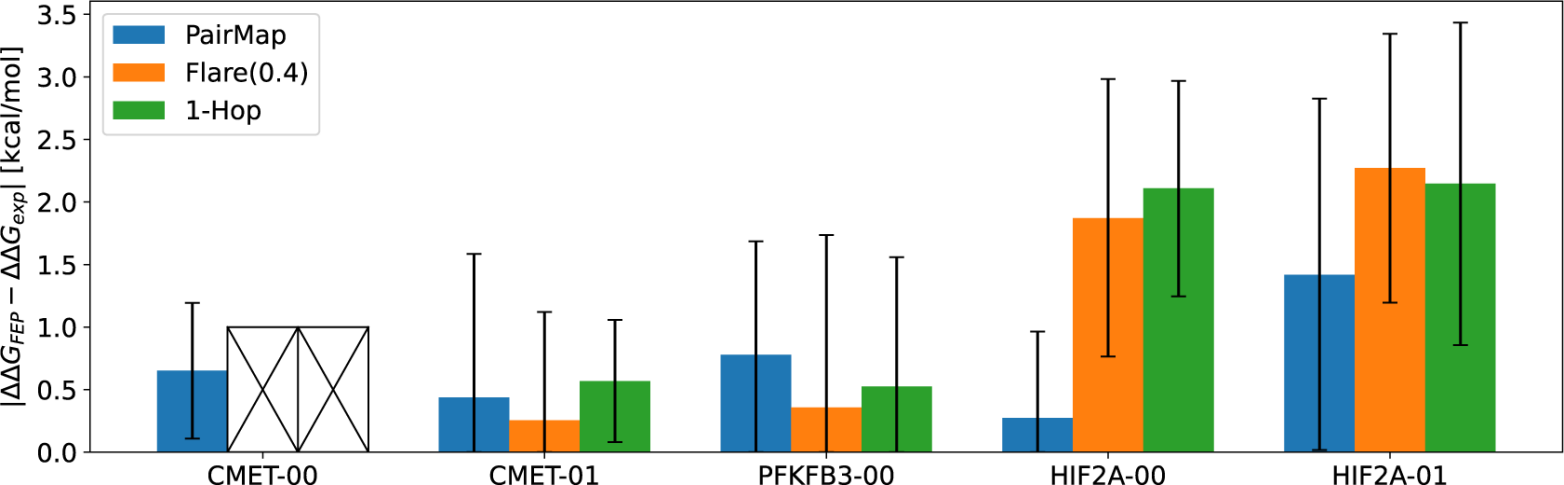
Results of ΔΔ*G*^*error*^ = |ΔΔ*G*_*exp*_ – ΔΔ*G*_*FEP*_| for the Merck data set.

In addition, we evaluated the performance of FEP calculations using the benchmark data set for relative free energy calculations made public by Schindler et al.^31^ (referred to as the Merck data set). The 5 selected compound pairs are shown in Figure 14. For these 5 pairs, we compared the FEP calculation accuracy among 3 methods: PairMap, Flare(0.4), and 1-Hop. Figure 15 shows the ΔΔ*G*^*error*^ for the Merck data set (Results for Δ*G* are shown in Figure S5). For CMET-00, the existing Flare FEP’s automatic intermediate generation could not insert appropriate intermediates, so 1-Hop and Flare(0.4) were considered a failure (see Figure S6). As shown in the figure, while the performance is roughly the same for cases like CMET-01 and PFKFB3–00, PairMap shows the best performance in the HIF2A-00 example. However, in the case of HIF2A-01, all methods show poor prediction accuracy. For HIF2A-00 and HIF2A-01, these involve compound pairs different from compound 224, where compound 30 has a lower Δ*G* and compound 43 has a higher Δ*G*. The difficulty in accurately predicting these differences is probably the cause of the poor prediction accuracy.

### PDE5a Scaffold Hopping Experiments

**Figure 16:**
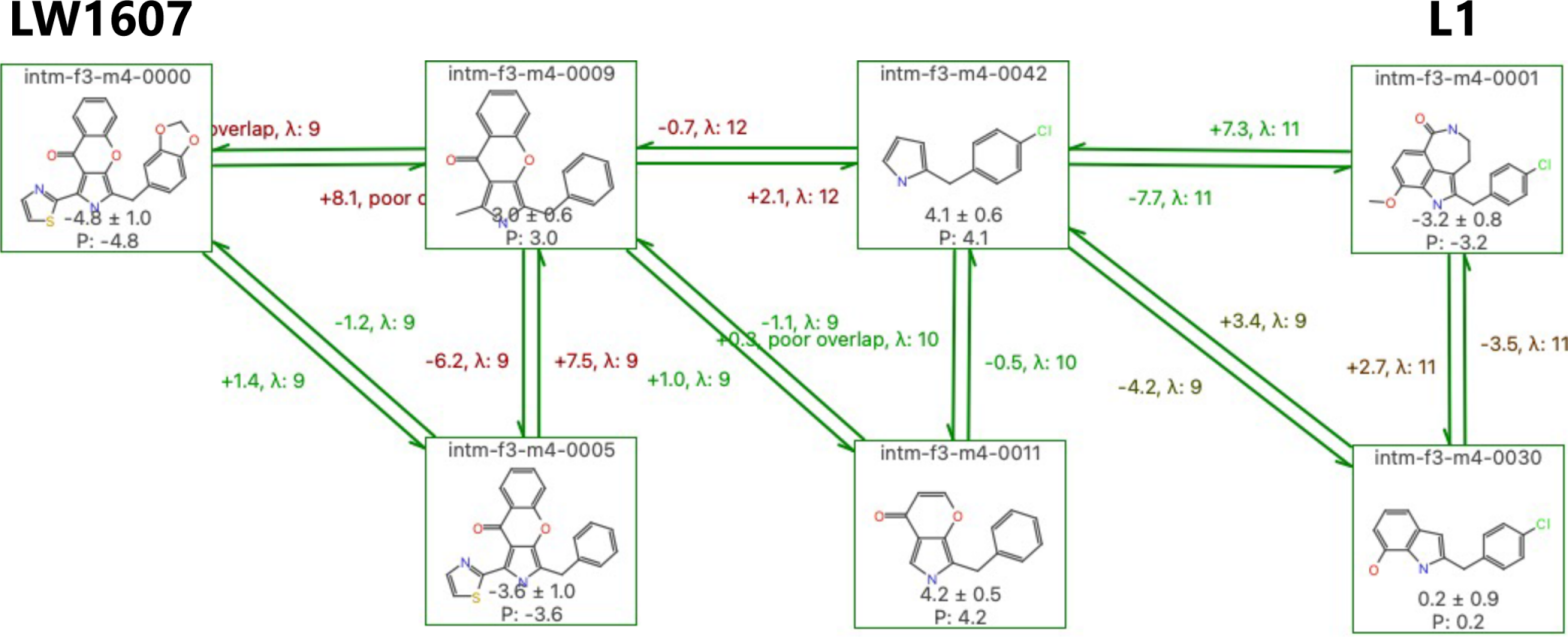
PairMap-derived perturbation map for PDE5a target inhibitors LW1607-L1.

Next, we examined a case study of actual scaffold hopping and tested the applicability of PairMap for FEP calculations with PDE5a inhibitors. In this experiment, we compared the simulation results with those of Wu et al.’s^23^ ABFEP-based scaffold hopping case study. Wu et al. performed scaffold hopping based on the pharmacophores of the potent PDE5a inhibitors Tadalafil^32^ and LW1607^33^ and designed the L1 structure. Further optimization of the L1 structure resulted in L12, a compound with potent affinity, high selectivity, and favorable pharmacological properties (refer to the figure for each compound). In this process, they used the ABFEP method to estimate that L1 was an inhibitor with Δ*G*_*FEP*_ = −10.98 kcal/mol compared to LW1607’s Δ*G*_*FEP*_ = −13.54 kcal/mol for the complex compound transformation from LW1607 to L1. In this experiment, we compared these results with RBFEP calculations using PairMap. The ligand-protein complex structure used was PDBID:7FAQ. Flare’s compound alignment function^9^ was used to generate the complex structures. FEP simulations were performed bidirectionally with *min*λ = 9. Figure 16 shows the ΔΔ*G*_*FEP*_ calculation results for LW1607-L1 using the perturbation map constructed by PairMap.

**Figure 17:**
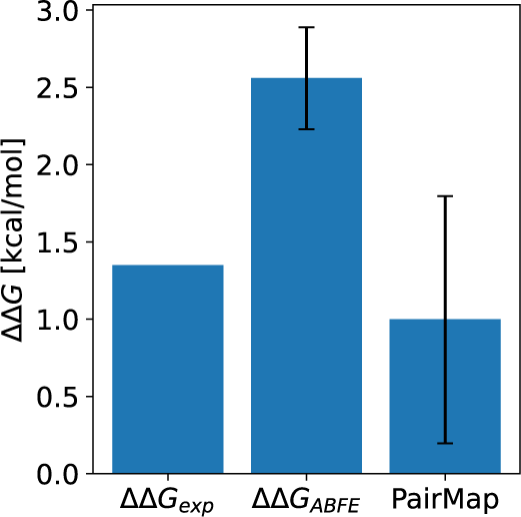
Results of ΔΔ*G* for PDE5a: LW1607-L1. The error bars were obtained from the standard deviation.

Figure 17 shows the results obtained after comparing the ΔΔ*G*_*FEP*_ values of LW1607-L1 for PDE5a with those obtained using the previous ABFEP method. The results indicate that the error in ΔΔ*G*_*exp*_ was smaller than that observed with ABFEP calculation. However, considering the large estimation error in RBFEP and the presence of errors of ±1 to 2 kcal/mol in many bidirectional links, while a definitive conclusion cannot be reached, the findings suggest at least comparable or better prediction accuracy. Given that the ABFEP method requires a larger computational cost than the RBFEP method, FEP calculations using PairMap can reasonably evaluate scaffold hopping with accuracy comparable to existing methods. By observing the perturbation map including intermediates, it is possible to analyze the partial structures that had a significant impact on prediction accuracy.

### Perturbation Map Construction for Congeneric Series

Finally, we examined the effect of reducing computational cost when intermediates were introduced for RBFEP calculations in a congeneric series. In this experiment, we targeted two congeneric series: the HIF2-α target from Schindler et al.’s^31^ FEP calculation benchmark, which has no charge changes, and the P38 target from Wang et al.’s data set. We created perturbation maps using 3 methods: no intermediate (building perturbation maps with Flare FEP’s default settings without introducing additional intermediates), Flare(0.6) (Flare FEP’s automatic intermediate generation with a link score threshold of 0.6), and PairMap, and compared their computational cost and accuracy.

Table 3 shows the statistics of perturbation maps for the HIF2-α and P38 data sets. For the HIF2-α data set of 42 compounds, the intermediate introduction increased to 54 compounds in Flare(0.6) and 46 compounds in PairMap. For the P38 data set with 34 compounds, the intermediate introduction increased to 42 compounds in Flare(0.6) and 39 compounds in PairMap. As a result, while the perturbation maps without intermediates had 4 bad edges with link scores below 0.4 in the HIF2-α data set and 2 in the P38 data set, both intermediate introduction methods removed these. However, edges with somewhat poor link scores between 0.4 and 0.6 remained even after introducing intermediates. In the HIF2-α data set, PairMap’s method most effectively reduced poor edges with just 4 intermediate introductions. Conversely, in the P38 data set, while PairMap resolved edges with link scores below 0.4, it increased the number of edges with scores below 0.6. Flare FEP reduced edges with scores below 0.6 by introducing more intermediates but significantly increased the total number of edges. Therefore, the P38 data set can be considered more challenging for eliminating poor link score edges.

**Table 3:** Statistics of Perturbation Map and FEP Simulations Performed on the HIF2-α Data Set

		# Nodes	# Links	# Links (*s* ≤ 0.6)	# Links (*s* ≤ 0.4)	Total λ*s*	Total λ*s* (λ*s* = 9)
HIF2-α	No Intermediates	42	58	9	4	1076	1044
	Flare(0.6)	54	78	4	0	1474	1404
	PairMap	46	63	3	0	1410	1134
P38	No Intermediates	34	48	11	2	982	918
	Flare(0.6)	42	64	10	0	1210	1152
	PairMap	39	55	15	0	1066	990

From a computational cost perspective, when the initial expected value before FEP calculation was λ*s* = 9, Flare(0.6) resulted in the highest cost, and while PairMap’s cost increase was minimized, there was no significant difference in the total actual λ*s* between Flare(0.6) and PairMap. This is because Flare FEP’s automatic λ*s* determination increased sampling for links that could have slower convergence. Since link scores do not necessarily correlate with actual FEP sampling requirements, improving link scores might not reduce FEP computational costs. Therefore, improving the link score metric itself might be necessary for more efficient perturbation map construction. On the other hand, for P38, the total actual λ*s* did not increase significantly with PairMap, indicating efficient intermediate introduction. Thus, while PairMap’s intermediate introduction method has limitations regarding link scores, it achieved its goal of introducing intermediates more efficiently than existing methods.

### Perturbation Map Construction Time for Congeneric Series

Table 4 shows the perturbation map construction time for the P38 and HIF2-α data sets. For both data sets, PairMap constructed perturbation maps several times faster than Flare(0.6), and when looking at perturbation map construction time alone, it was significantly faster. This is likely because Flare(0.6) reconstructs the perturbation map from scratch each time an intermediate is introduced. Therefore, PairMap can construct perturbation maps in practical time even for larger congeneric series compared to existing methods. However, as mentioned earlier, when targeting several hundred compound series, the number of links requiring intermediates may increase, potentially causing execution time to increase linearly with the number of necessary intermediates.

**Table 4:** Perturbation Map Construction Time (Seconds) for Congeneric Series^a^

Target	Flare(0.6)	PairMap
P38	1277	656(75)
HIF2-α	4222	806(83)

a Flare(0.6) includes not only the time required for perturbation map construction but also the total time for FEP project set up. For PairMap, the sum of perturbation map construction and FEP project set up times is shown for comparison, with the time required for perturbation map construction shown in parentheses.

### FEP Calculation Results for Congeneric Series

**Figure 18:**
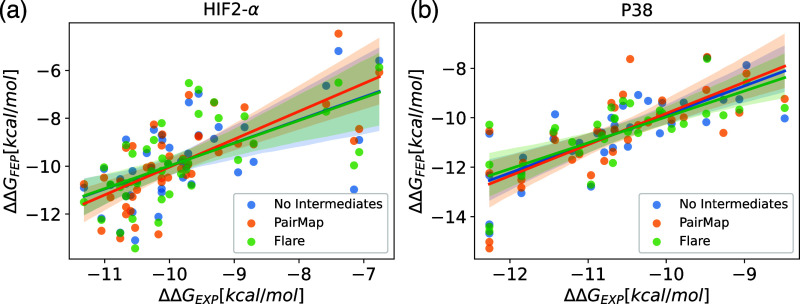
Scatter plots of ΔΔ*G*_*exp*_ versus ΔΔ*G*_*FEP*_ for (a) HIF2-α data set and (b) P38 data set.

Table 5 shows the results of FEP simulations for the two congeneric series. It shows the following metrics between ΔΔ*G*_*exp*_ and ΔΔ*G*_*FEP*_ : Mean Absolute Error (MAE), Pearson’s R-squared value (Pearson’s *R*^2^), and Kendall’s τ value. For the HIF2-α data set, PairMap showed the best results for Pearson *R*^2^ and Kendall’s τ, while MAE was similar across all methods (Figure 18a). Therefore, in some cases, eliminating poor links through intermediate introduction contributed to improved accuracy. On the other hand, for the P38 data set, there were no significant differences in correlations among the methods. Regarding MAE, PairMap showed slightly higher values, likely due to increased errors in some calculations (Figure 18b). This might be because the number of edges with link scores below 0.6 increased while trying to remove edges with scores below 0.4. In the case of P38, neither Flare’s method nor PairMap showed an improvement in accuracy worth the increased computational cost, indicating the need for further improvement of the intermediate introduction method.

**Table 5:** Results of FEP Simulations on the HIF2-α and P38 Data Sets

		MAE [kcal/mol]	Pearson’s *R*^2^	Kendall’s τ
	No Intermediates	1.06 ± 0.20	0.39 ± 0.11	0.45 ± 0.09
HIF2-α	Flare(0.6)	1.06 ± 0.18	0.37 ± 0.10	0.48 ± 0.08
	PairMap(0.6)	0.99 ± 0.27	0.52 ± 0.17	0.57 ± 0.11
	No Intermediates	0.77 ± 0.16	0.58 ± 0.10	0.64 ± 0.09
P38	Flare(0.6)	0.75 ± 0.26	0.53 ± 0.16	0.58 ± 0.15
	PairMap(0.6)	0.88 ± 0.27	0.54 ± 0.15	0.62 ± 0.12

## Conclusions

In this paper, we proposed PairMap, a method for constructing perturbation maps using intermediates to appropriately perform RBFEP calculations for complex compound transformations, to enhance the convenience of lead optimization using FEP. Exhaustive intermediate generation and optimal intermediate paths can achieve simpler transformations with minimal introduction of intermediates compared to existing methods. Existing Flare FEP’s automatic intermediate generation approaches of iteratively introducing intermediates may fail to find simpler paths that can be achieved with fewer intermediate introductions; hence, it is important to consider intermediates exhaustively, as demonstrated by this method. Furthermore, the proposed method can improve the prediction accuracy of ΔΔ*G*_*FEP*_ compared to Flare FEP’s methods by constructing perturbation maps that consider cyclicity, distance, and other factors. The PDE5a scaffold hopping experiment suggested that this method can be an option for practical use cases of FEP calculations. Furthermore, we verified the effect of introducing intermediates with PairMap in the conventional FEP use case of calculating binding affinities for the congeneric series. Introducing intermediates in the perturbation map in the proposed method can solve more reasonably complex transformations than previous approaches.

In terms of enhancing accuracy for complex compound transformations, this method may be particularly effective only for FEP calculations based on the Single Topology method.^19,34^ As shown in Figure S8, the FEP simulations in OpenFE,^35^ based on the hybrid topology approach,^36^ showed no improvement in accuracy with the introduction of intermediates; notably, the accuracy was worse than that in the Flare FEP case. However, the introduction of intermediates to reduce computational cost in FEP calculations for a series is expected to have some effect regardless of the topology method. Although this method assumes that the compounds have a common scaffold, it is challenging when the scaffold varies significantly and atom mapping is not available, which is a crucial limitation of proposed method. In such cases, it is appropriate to use methods such as ABFEP or the separated topology method^37,38^ which calculate relative binding free energies not requiring atom mapping. Additionally, the proposed method can currently target only the introduction of intermediates between compounds with no change in net charge. Therefore, in future studies, we would like to investigate methods to introduce intermediates in cases that involve changes in the net charge^39,40^ between input compounds. Moreover, incorporating the proposed method into exploratory approaches such as recent study involving lead optimization through active learning^41−43^ with FEP^44−49^ would help expand the searchable compound space and streamline overall computations. Furthermore, PairMap method was shown to be more practical than existing methods in terms of execution time.

The series of frameworks proposed in this method support lead optimization in various situations when applying FEP calculations in practice.

## Data Availability

The PairMap code and the dataset used in this manuscript are available on github; https://github.com/ohuelab/PairMap.

## Supporting Information Available

Results of ΔΔ*G*_*FEP*_ on the Wang-9 dataset (compared with 1-Hop); results of ΔΔ*G*_*FEP*_ on the Wang-9 dataset (compared with Flare’s automatic intermediate introduction); results of ΔΔ*G*_*FEP*_ on the Wang-9 dataset (comparison at MAX_DIST); perturbation map after calculation with PairMap(3) for PTP1B-01; results of ΔΔ*G*_*FEP*_ on the Merck dataset; results of perturbation map using Flare’s automatic intermediate generation for CMET-00; a part of the perturbation map with automatic intermediate generation by Flare FEP for HIF2-α dataset (threshold 0.6); results of RBFEP calculations using OpenFE on the Wang-9 dataset (Figures S1–S8) (PDF)
