# Supplementary material for: PairMap: An Intermediate Insertion Approach for Improving the Accuracy of Relative Free Energy Perturbation Calculations for Distant Compound Transformations
Source: J Chem Inf Model. 2025 Jan 13;65(2):705–21. doi: 10.1021/acs.jcim.4c01634 (PMC11776053; doi:10.1021/acs.jcim.4c01634)
Supplement: Supplementary file 1 — ci4c01634_si_001.pdf [file ci4c01634_si_001.pdf]

Supporting Information: PairMap: An Intermediate Insertion Approach for Improving the Accuracy of Relative Free Energy Perturbation Calculations for Distant Compound Transformations

Kairi Furui,[†] Takafumi Shimizu,[‡] Yutaka Akiyama,[¶] S. Roy Kimura,[‡] Yoh Terada,[‡] and Masahito Ohue[*,†]

†*Department of Computer Science, School of Computing, Institute of Science Tokyo, Yokohama 226-8501, Japan*

‡*Alivexis, Inc., Tokyo 105-0004, Japan*

¶*Department of Computer Science, School of Computing, Institute of Science Tokyo, Tokyo 152-8550, Japan*

E-mail: ohue@comp.isct.ac.jp

Phone: +81 (0)45 924 5522. Fax: +81 (0)45 924 5523

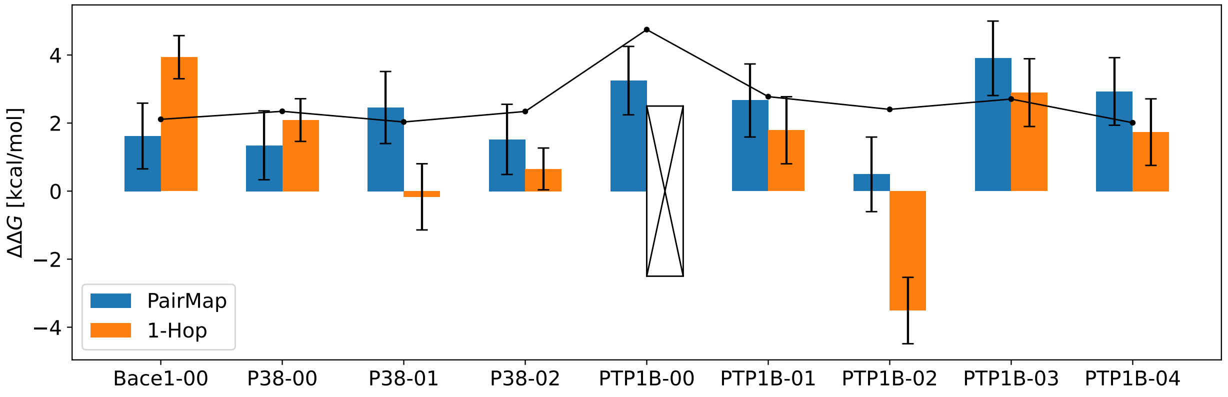

Figure S1: Results of $\Delta\Delta G_{FEP}$ on the Wang-9 dataset (comparison of PariMap and 1-Hop). A cross in the bar chart means that the $\Delta\Delta G$ calculation failed.

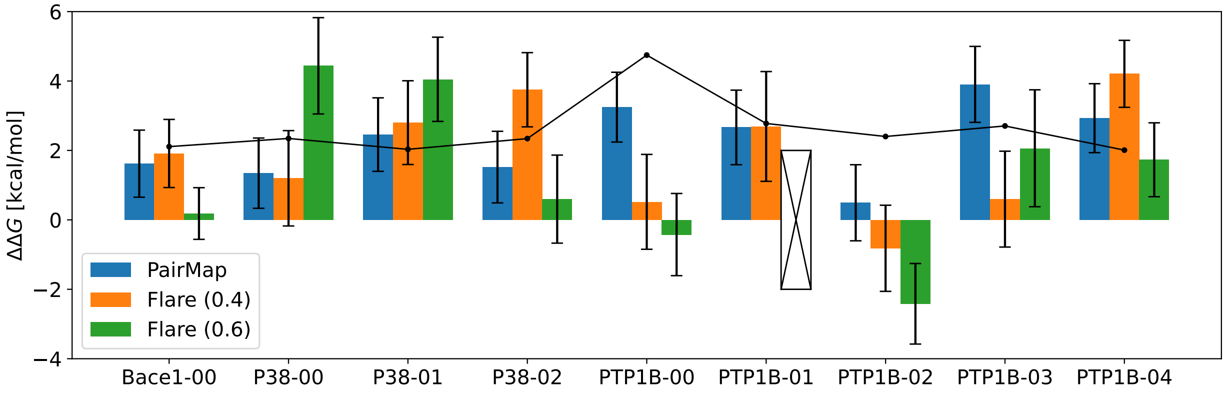

Figure S2: Results of $\Delta\Delta G_{FEP}$ on the Wang-9 dataset (comparison of PariMap and Flare's automatic intermediate introduction). A cross in the bar chart means that the $\Delta\Delta G$ calculation failed.

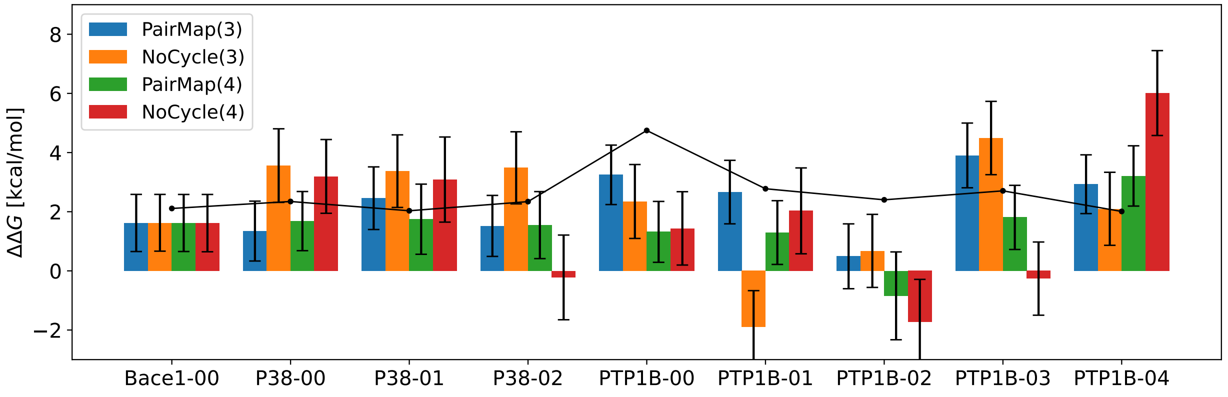

Figure S3: Results of $\Delta\Delta G_{FEP}$ on the Wang-9 dataset (comparison at MAX_DIST).

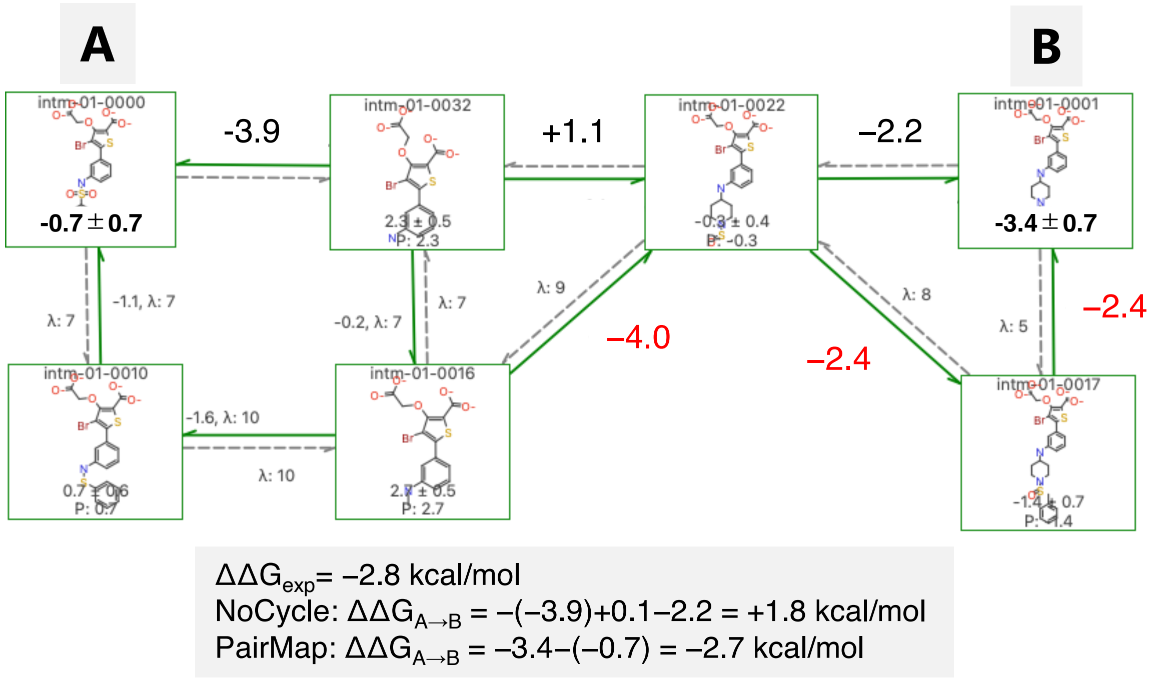

Figure S4: Perturbation map calculated with PairMap(3) for PTP1B-01. The values on each link represent $\Delta\Delta G$ (kcal/mol). NoCycle(3) is calculated along the shortest path from A to B, and its prediction is opposite to the experimental value. The red links show more negative $\Delta\Delta G$ values compared to their corresponding links of optimal path, predicting a lower ΔG for Ligand B. This compensatory effect improves the overall prediction accuracy in PairMap(3).

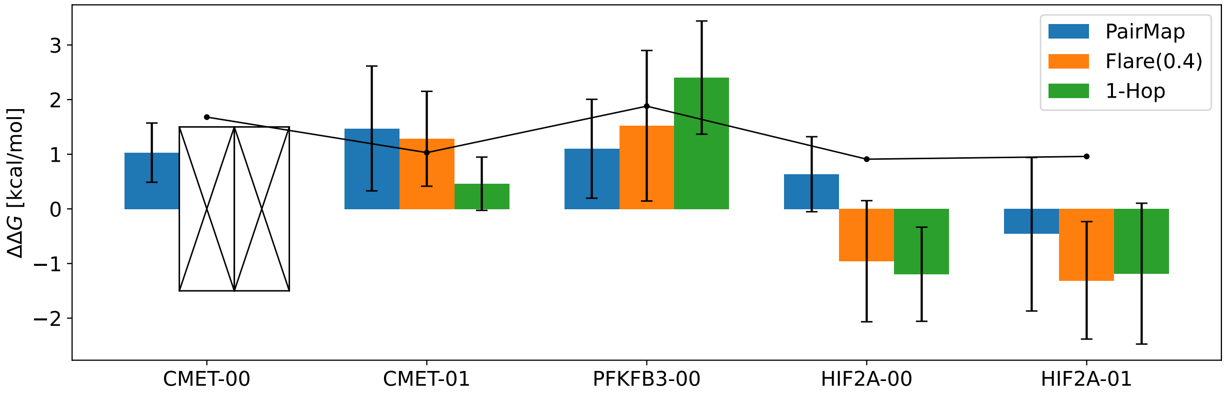

Figure S5: Results of $\Delta\Delta G_{FEP}$ on the Merck dataset. A cross in the bar chart means that the $\Delta\Delta G$ calculation failed.

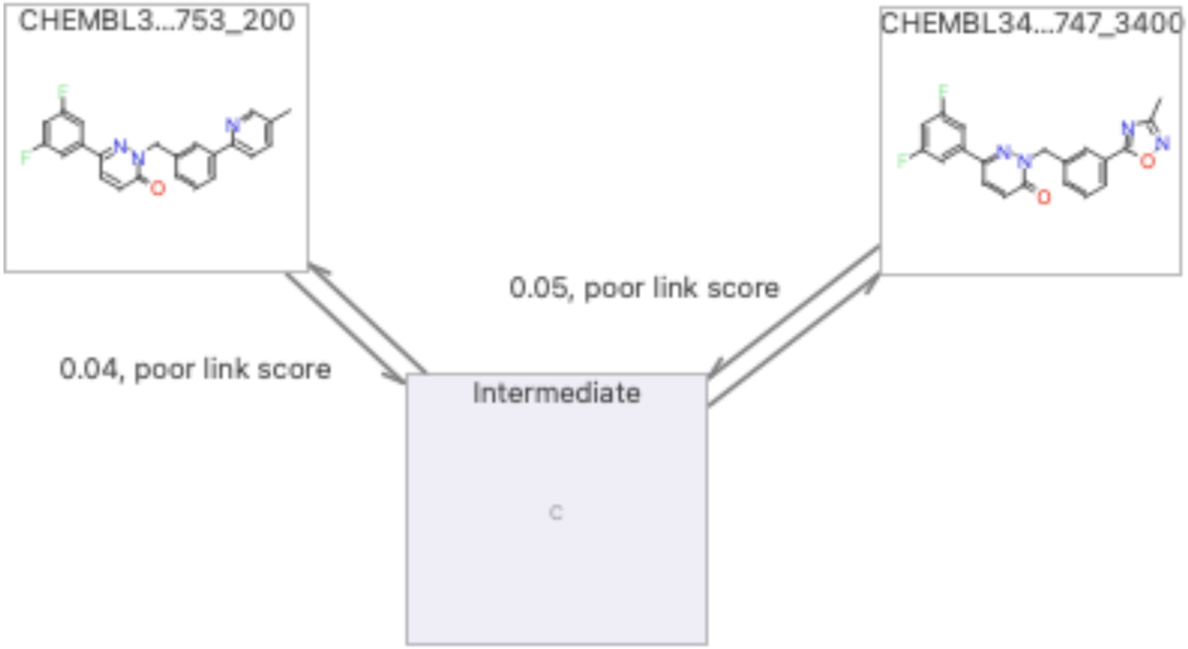

Figure S6: Results of perturbation map using Flare's automatic intermediate generation for CMET-00. It was considered a failure because Flare FEP could only produce an intermediate with only one carbon atom.

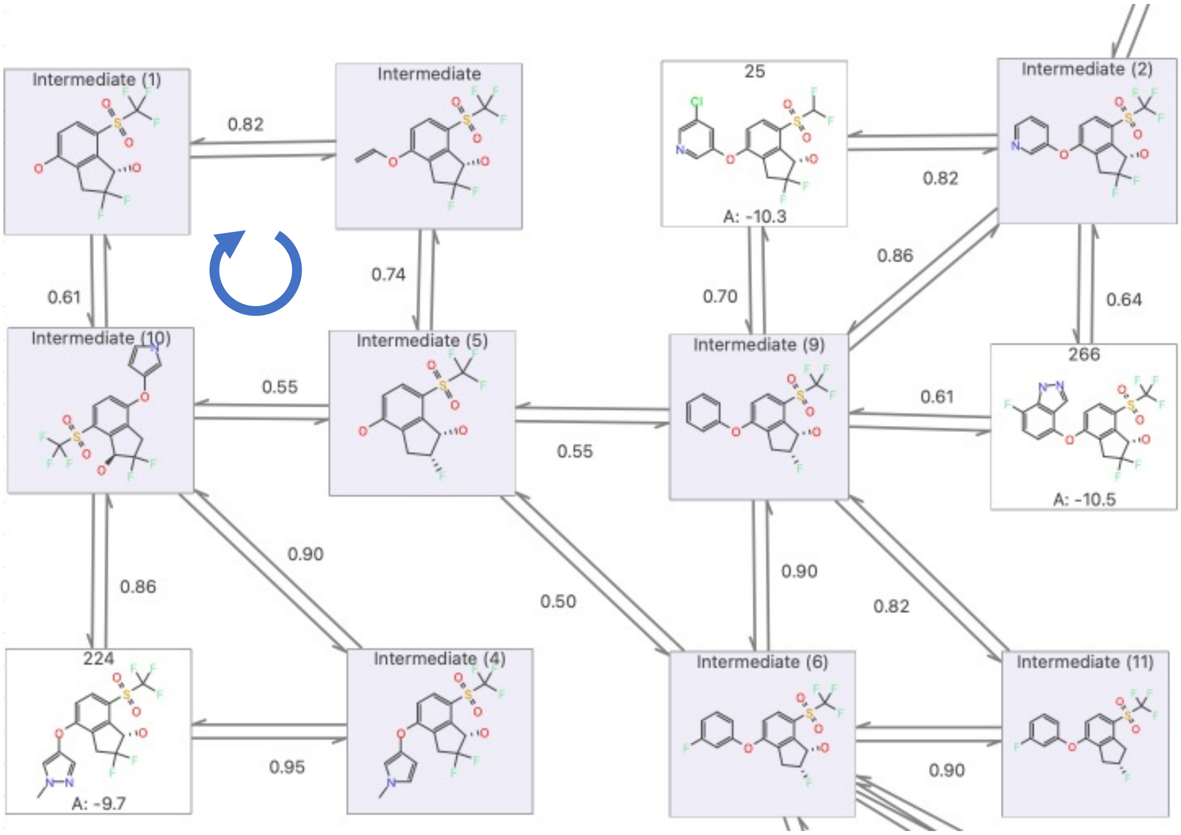

Figure S7: A part of the perturbation map with automatic intermediate generation by Flare FEP for HIF2-α dataset (threshold 0.6). The cycle indicated by the arrow is composed only of intermediates, and can be redundant in FEP calculations for derivative series.

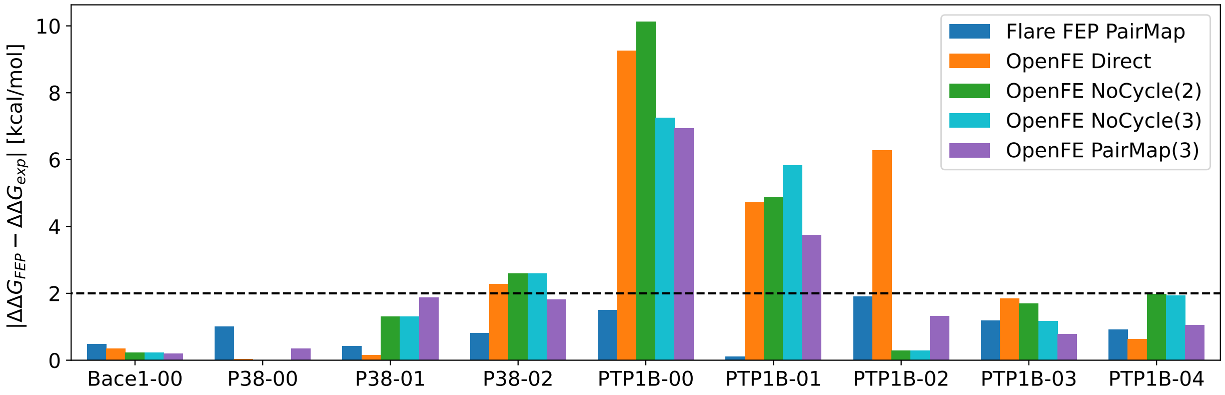

Figure S8: Results of RBFEP calculations using OpenFE on the Wang-9 dataset. No error bars are shown because OpenFE did not provide uncertainty values. Flare FEP is more accurate overall, as several errors above 2 kcal/mol are observed with OpenFE.
